# Novel aspect of neprilysin in kidney fibrosis via ACSL4‐mediated ferroptosis of tubular epithelial cells

**DOI:** 10.1002/mco2.330

**Published:** 2023-07-14

**Authors:** Weijing Lai, Rongshuang Huang, Bo Wang, Min Shi, Fan Guo, Lingzhi Li, Qian Ren, Sibei Tao, Ping Fu, Liang Ma

**Affiliations:** ^1^ Department of Nephrology Kidney Research Institute West China Hospital of Sichuan University Chengdu China; ^2^ Department of Nephrology Clinical Medical College and The First Affiliated Hospital of Chengdu Medical College Chengdu China

**Keywords:** ACSL4, ferroptosis, kidney fibrosis, neprilysin, sacubitrilat, tubular epithelial cell

## Abstract

Although inhibition of neprilysin (NEP) might be a therapeutic strategy with the potential to improve the outcome of chronic kidney disease (CKD), the versatile function of NEP with its mechanism remains obscure in kidney fibrosis. In the study, we found that NEP was abnormally increased in tubular epithelial cells of CKD patients, as well as unilateral ureteral obstruction and adenine diet‐induced mice. Treatment with a United States Food and Drug Administration‐approved NEP inhibitor Sacubitrilat (LBQ657) could alleviate ferroptosis, tubular injury, and delay the progression of kidney fibrosis in experimental mice. Similarly, genetic knockdown of NEP also inhibited tubular injury and fibrosis in transforming growth factor (TGF)‐β1 ‐induced tubular cells. Mechanically, NEP overexpression aggravated the ferroptotic and fibrotic phenotype, which was restored by acyl‐CoA synthetase long‐chain family member 4 (ACSL4) knockdown. The NEP silencing attenuated TGF‐β1‐induced tubular cell ferroptosis and was exacerbated by ACSL4 overexpression. Collectively, for the first time, a novel aspect of NEP was explored in kidney fibrosis through ACSL4‐mediated tubular epithelial cell ferroptosis. Our data further confirmed that NEP inhibition exerted a promising therapeutic against fibrotic kidney diseases.

## Graphical abstract

Abnormally elevated neprilysin (NEP) could aggravate kidney fibrosis via acyl‐CoA synthetase long‐chain family member 4 (ACSL4)‐mediated tubular epithelial cell ferroptosis. Pharmacological or genetic inhibition of NEP could exert a promising therapeutic against fibrotic kidney diseases.

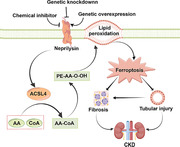

## 1 INTRODUCTION

Chronic kidney disease (CKD) affected almost 10% of the global population,^1^ and the mortality rate increased by 41.5% between 1990 and 2017,^2^ which is expected to be the fifth leading cause of premature death worldwide by 2040.^3^ As a critical public health problem worldwide, CKD severely affects individuals, their families, and the community at large. Kidney fibrosis is the most common progressive process in CKD, deteriorating kidney function and eventually leading to end‐stage kidney disease.^4^ Although tremendous efforts have been made to fight against kidney fibrosis, there is still a lack of therapeutic options. Therefore, it is of great importance to explore the pathogenesis of kidney fibrosis and discover new drug targets.

Neprilysin, also known as neutral endopeptidase (NEP), membrane metalloendopeptidase, and neutrophil antigen cluster differentiation antigen 10 (CD10), was discovered as a single‐pass type II membrane protein in 1970.^5^ NEP is a member of the M13 family of zinc endopeptidases, whose genes are located on chromosome 3q25.2.^5^, ^6^ And is expressed primarily in the brush border membrane of the proximal renal tubule, but also in the brain, lung, heart, testes, pancreas, intestine, and adipose tissues.^7^, ^8^ As a multisubstrate enzyme, NEP hydrolyses peptides up to about 50 amino acids long.^9^ With extensive application of the NEP inhibitor, its renoprotective function has been gradually recognized. A series of studies demonstrated that NEP inhibition enhanced the activity of natriuretic peptide systems causing natriuresis, diuresis, vasodilatation, and inhibition of renin‐angiotensin system (RAS), which could act as a potentially beneficial counter‐regulatory system in RAS activation states such as chronic heart failure and CKD.^10^, ^11^, ^12^ However, the versatile function of NEP with its mechanism remains obscure in kidney fibrosis of CKD.

(Ferroptosis is an iron‐dependent‐regulated cell death, which is characterized by lipid peroxidation and the accumulation of lethal lipid reactive oxygen species (ROS) that leads to cell death.^13^ Acyl‐CoA synthetase long‐chain family member 4 (ACSL4), an activator of polyunsaturated fatty acids, plays an important role in lipid peroxidation and has been considered the main biomarker and trigger of ferroptosis.^14^ Recently, an ever growing number of data has highlighted the role of ferroptosis in kidney fibrosis. In unilateral ureter obstruction (UUO), 5/6 nephrectomy, ischemia/reperfusion injury (IRI) or folic acid‐induced fibrotic kidneys, evidence of ferroptosis has been found, including iron overload, oxidative stress, lipid peroxidation, mitochondrial defects, and expression of ferroptosis markers, while inhibition of ferroptosis improved kidney injury and fibrosis.^15^, ^16^, ^17^ These phenomena were further verified in renal tubular epithelial cells (RTEC) in vitro, such as rat proximal RTECs (NRK‐52E) stimulated by transforming growth factor (TGF)‐β1 and human renal proximal tubular (HK‐2) cells stimulated by high glucose.^18^, ^19^

However, the unexplorable mechanism that NEP regulating ferroptosis in CKD is still unknown. In this study, we systematically revealed that NEP elevation contributed to the progression of kidney fibrosis, and discovered that inhibition of NEP could alleviate kidney fibrosis through ACSL4‐mediated tubular epithelial cell ferroptosis, suggesting that inhibition of NEP may be a promising therapeutic against fibrotic kidney diseases.

## 2 RESULTS

### 2.1 Renal NEP was abnormally elevated in CKD patients and mice

**FIGURE 1:**
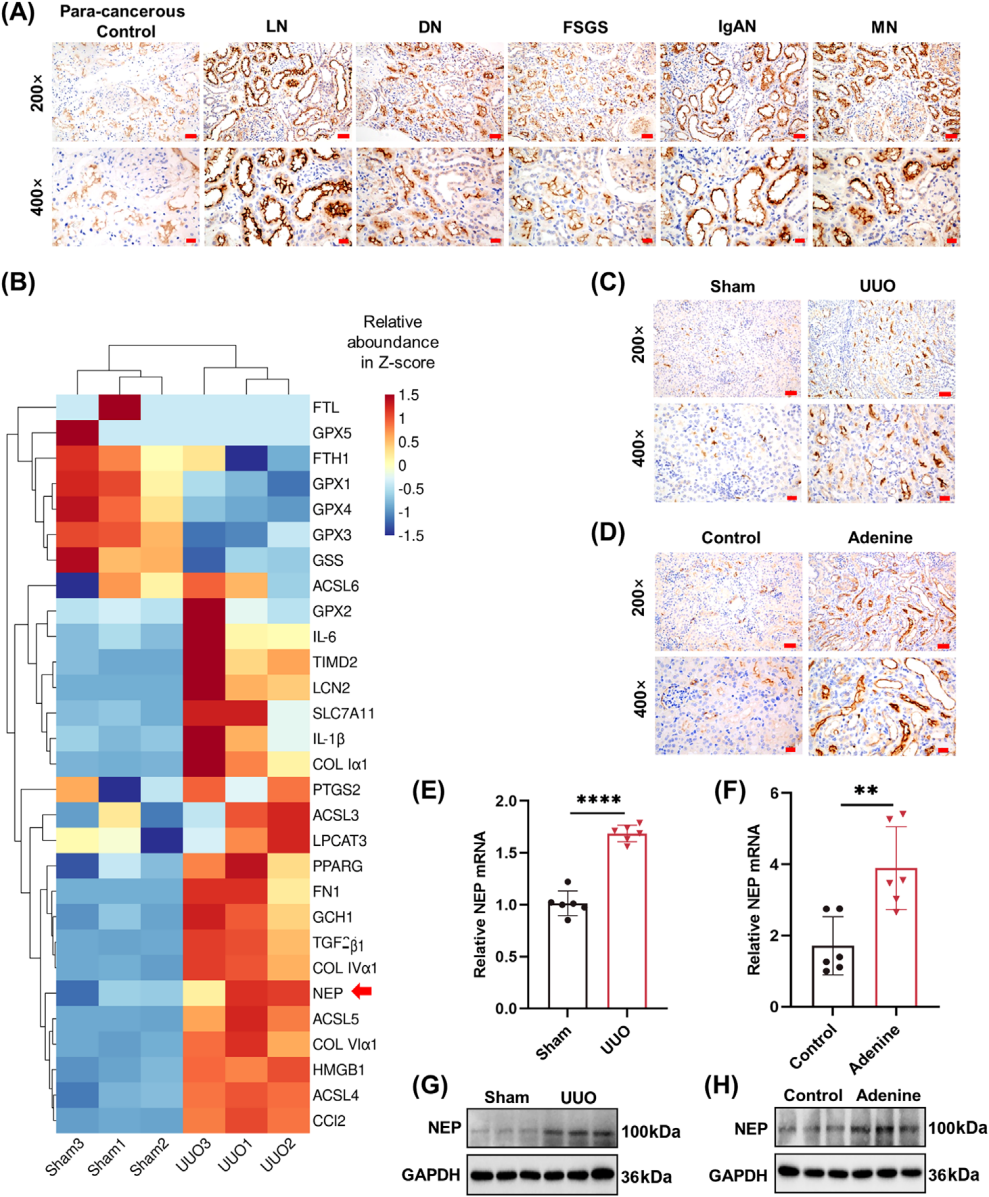
Renal neprilysin (NEP) was abnormally elevated in CKD patients and mice. (A) Immunochemistry staining of NEP in human kidney sections (×200, scale bar = 50 μm; ×400, scale bar = 20 μm). LN, lupus nephritis; DN, diabetic Nephropathy; FSGS, focal segmental glomerulosclerosis; IgAN, IgA nephropathy; MN, membranous nephropathy. (B) Heatmap of differentially expressed genes in the kidneys of mice between the sham and UUO group (*n* = 3). (C and D) Immunochemistry staining of NEP in mouse kidney sections (×200, scale bar = 50 μm; ×400, scale bar = 20 μm). (E and F) Relative levels of NEP mRNA normalized by GAPDH. (G and H) Relative levels of the NEP protein normalized by GAPDH. ****p < 0.0001, **p < 0.01.

NEP expression was abnormally elevated in kidney biopsies of CKD patients due to different primary diseases, such as lupus nephritis, diabetic nephropathy, focal segmental glomerulosclerosis, IgA nephropathy, and membranous nephropathy (Figures 1A and S1A). Furthermore, the increased expression of NEP mRNA and protein in the fibrotic kidneys of UUO mice was confirmed by transcriptomic analysis of RNA‐sequencing (Figure 1B), immunohistochemical staining (Figures 1C and S1B), RT‐PCR (Figure 1E), and Western blotting (Figures 1G and S1D). Similarly, the protein level (Figures 1D, H and S1C, E) and the mRNA level (Figure 1F) of NEP were also upregulated in the injured kidneys of CKD mice induced by adenine diet. Further analysis of kidney transcriptomics of UUO mice suggested that increased NEP transcription was potentially related to these gene expression involved in fibrosis, ferroptosis, inflammation, and tubular injury (Figure 1B).

### 2.2 Pharmacological inhibition of NEP alleviated kidney fibrosis in mice induced by UUO and adenine diet

To confirm whether NEP inhibition provides a protective effect on kidney fibrosis, we applied a United States Food and Drug Administration‐approved drug NEP inhibitor *Sacubitrilat* (LBQ657) in the two experimental mouse models induced by UUO and adenine diet, respectively. First, we evaluated the activity and expression of NEP in serum and kidneys and found that serum and renal NEP activity was elevated in UUO mice and decreased significantly after the LBQ657 intervention (Figures S2A and B). The level of bradykinin (BK), a specific substrate of NEP, changed with the activity of NEP (Figures S2C and D). We also verified the inhibitory effect of LBQ657 on the elevation of NEP by using immunohistochemical staining (Figures S2E and F), RT‐PCR (Figures S2G and H), and Western blotting (Figures S2I and J).

**FIGURE 2:**
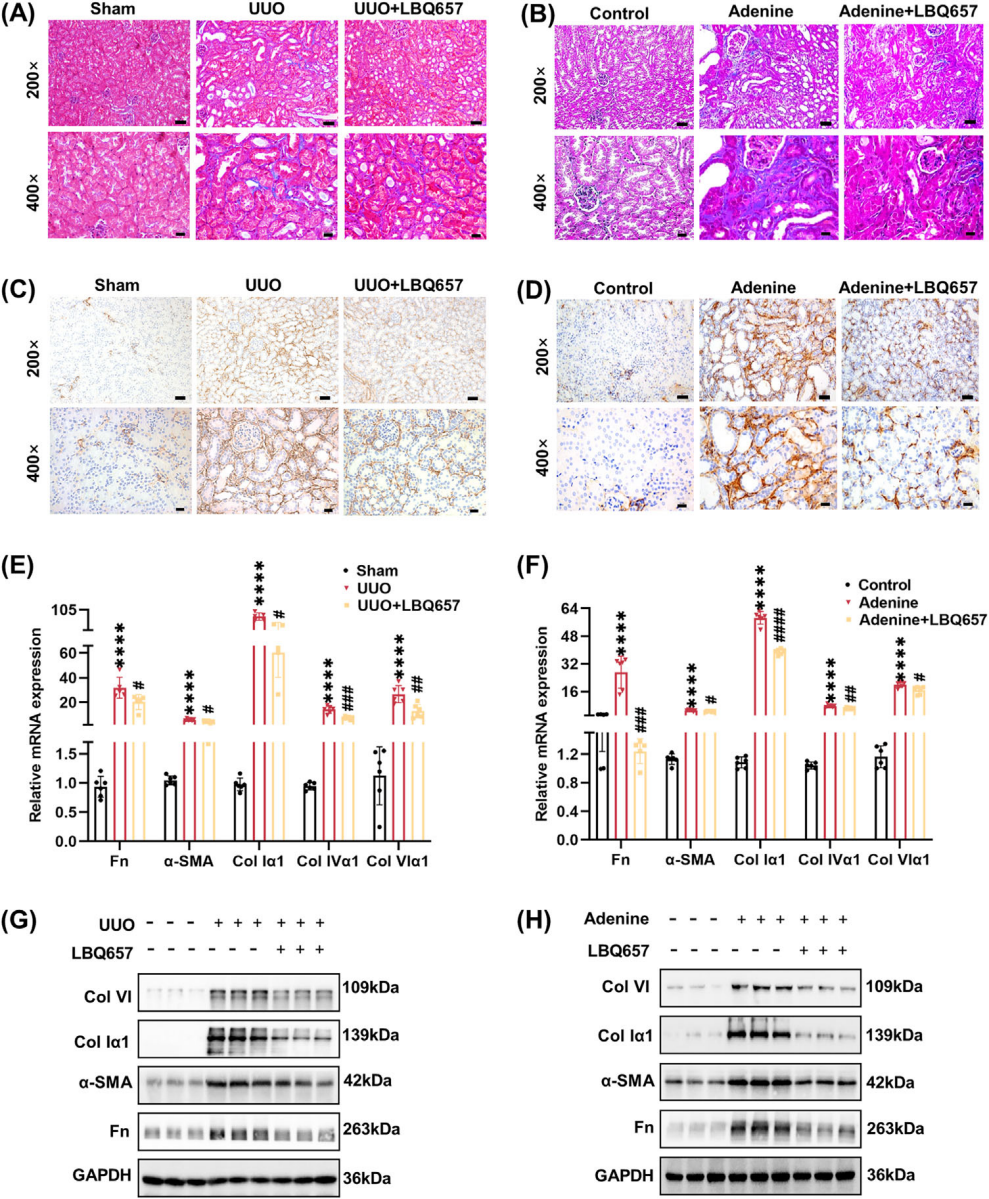
Pharmacological inhibition of neprilysin alleviated kidney fibrosis in mice induced by UUO and adenine diet. (A and B) Masson's trichrome staining of mouse kidney tissues (×200, scale bar = 50 μm; ×400, scale bar = 20 μm). (C and D) Immunochemistry staining of α‐SMA in mouse kidney sections (×200, scale bar = 50 μm; ×400, scale bar = 20 μm). (E and F) RT‐PCR was performed to detect the mRNA level of Fn, α‐SMA, Col Iα1, and Col VI. (G and H) Western blot analysis was performed to detect protein expression of Fn, α‐SMA, Col Iα1, and Col VI. ****p < 0.0001, versus sham or control. ^####^ *p <* 0.0001, ^###^ *p <* 0.001, ^##^ *p <* 0.01, ^#^ *p <* 0.05, versus UUO or Adenine.

Next, we examined the pathological changes in the injured kidneys of LBQ657‐treated mice. The result of Masson's trichrome staining showed less collagen deposition in the kidneys of mice treated with LBQ657 compared with those of UUO and adenine diet‐induced groups, respectively (Figures 2A, B and S3A, B). Since α‐smooth muscle actin (α‐SMA) is a characteristic marker of activated fibroblasts, we also detected that α‐SMA was upregulated in the kidneys of mice induced by UUO and adenine diet, while the corresponding increase was suppressed by administration of LBQ657 by immunohistochemical staining (Figures 2C, D and S3C, D). Similarly, NEP inhibition also suppressed key fibrotic gene mRNA levels such as fibronectin (Fn), α‐SMA, collagen Iα1 (Col Iα1), collagen IVα1 (Col IVα1), and collagen VIα1 (Col VIα1) (Figures 2E and F), which these trends in protein level were also confirmed by Western blotting (Figures 2G, H and S3E, F). Together, these results indicated that NEP inhibition might exert a notable protective effect against kidney fibrosis.

### 2.3 NEP inhibition alleviated tubular injury and kidney inflammation in UUO and adenine diet‐induced mice

**FIGURE 3:**
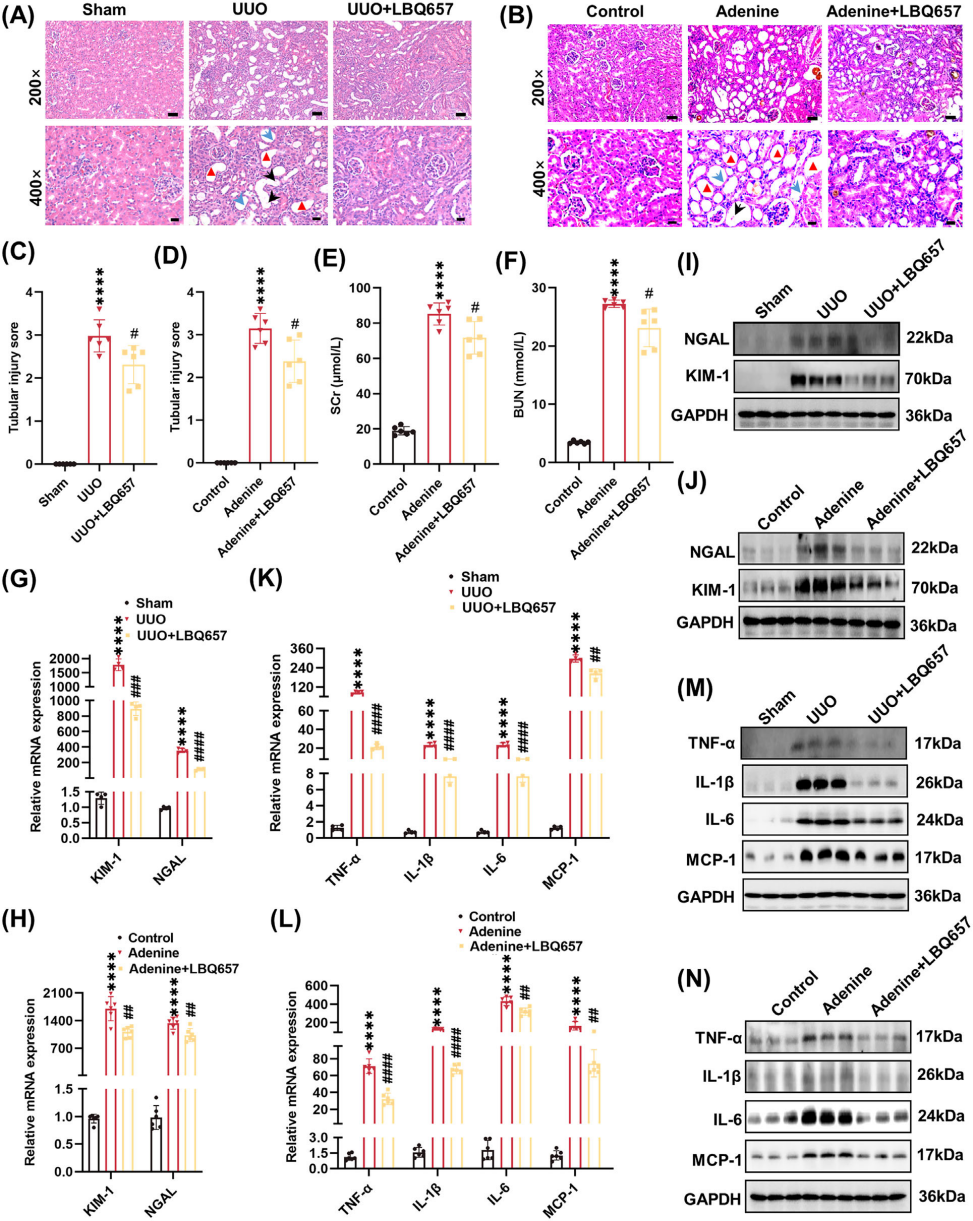
Neprilysin inhibition alleviated tubular injury and kidney inflammation in mice induced by UUO and adenine diet. (A and B) HE staining of kidney tissues (×200, scale bar = 50 μm; ×400, scale bar = 20 μm). Red triangle: tubule dilation; black arrow: necrosis and shedding of epithelial cells; blue arrow: disappearance of the tubule brush border. (C and D) Tubular injury scores for kidney tissues. (E) Serum creatinine (Scr) in different groups of mice. (F) Blood urea nitrogen (BUN) in different groups of mice. (G and H) RT‐PCR detected the mRNA level of KIM‐1 and NGAL. (I and J) Western blot analysis detected the expression of KIM‐1 and NGAL proteins. (K and L) RT‐PCR detected the mRNA level of MCP‐1, IL‐6, IL‐1β, and TNF‐α. (M and N) Western blot analysis detected protein expression of MCP‐1, IL‐6, IL‐1β and TNF‐α. ****p < 0.0001, versus sham or control. ^####^ *p <* 0.0001, ^###^ *p <* 0.001, ^##^ *p <* 0.01, ^#^ *p <* 0.05, versus UUO or adenine.

Tubular injury is a hallmark of CKD and a primary cause of kidney fibrosis.^20^ Renal histopathological changes were examined by hematoxylin–eosin (HE) staining, and we found that inhibition of NEP significantly alleviated loss of brush borders and dilatation of tubular epithelia (Figures 3A and B). Furthermore, NEP inhibition markedly decreased cumulative tubular injury scores in UUO mice (from 2.98 ± 0.34 to 2.32 ± 0.41) and adenine diet‐induced mice (from 3.15 ± 0.32 to 2.38 ± 0.45) (Figures 3C and D), respectively. Consistently, the levels of mRNA and protein of tubular injury marker *Havcr1* (kidney injury molecule 1, KIM‐1) and *Lcn2* (neutrophil gelatinase‐associated lipocalin, NGAL) also decreased by LBQ657 treatment (Figures 3G–J and S4A, B) in the kidneys of fibrotic mice. After administration of the adenine diet, serum creatinine levels (Scr) and blood urea nitrogen (BUN) levels were increased from 18.95 ± 2.11 to 85.23 ± 5.71 μmol/L and 3.48 ± 0.21 to 27.24 ± 0.58 mmol/L, while inhibition of NEP reduced Scr to 71.75 ± 8.44 μmol/L and BUN to 23.12 ± 2.95 mmol/L (Figures 3E and F). These results suggested that NEP blockade alleviated renal tubular injury in kidneys of UUO and adenine diet‐induced mice and exhibited a protective effect on renal function.

Proinflammatory cytokines such as monocyte chemoattractant protein–1 (MCP‐1), interleukin (IL)‐6, IL‐1β, and tumor necrosis factor alpha (TNF‐α) are increased in the fibrotic kidney,^21^ and the inflammatory response is a critical component of tubule‐mediated kidney fibrosis.^22^, ^23^ In the present study, the levels of mRNA and protein of MCP‐1, IL‐6, IL‐1β, and TNF‐α in kidney tissues were significantly elevated in mice induced by UUO and adenine diet, while inhibition of NEP suppressed the increase of these corresponding factors (Figures 3K–N and S4C, D). These data suggested that inhibition of NEP alleviated inflammation in the kidneys of UUO and adenine diet‐induced mice.

### 2.4 NEP inhibition alleviated tubular injury and fibrotic phenotype in TGF‐β1‐induced tubular epithelial cells

**FIGURE 4:**
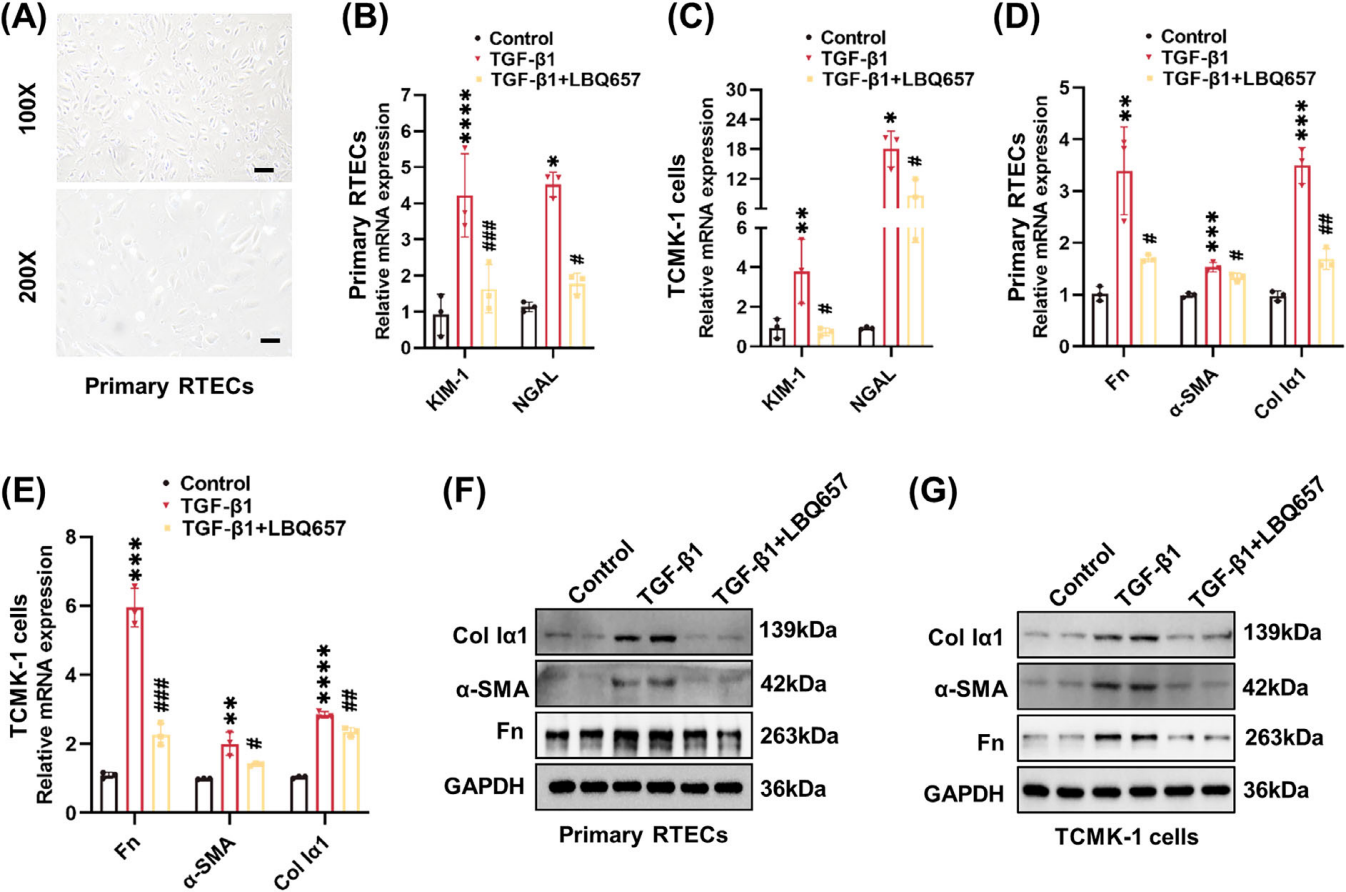
Neprilysin inhibition alleviated tubular injury and fibrotic phenotype in TGF‐β1‐stimulated tubular epithelial cells. (A) Isolation of mouse primary tubular epithelial cells (×100, scale bar = 100 μm; ×200, scale bar = 50 μm). (B and C) RT‐PCR was performed to detect the mRNA level of KIM‐1 and NGAL. (D and E) RT‐PCR was performed to detect the mRNA expression of Fn, α‐SMA, and Col Iα1. (F and G) Western blot analysis was performed to detect protein expression of Fn, α‐SMA, and Col Iα1. ****p < 0.0001, ***p < 0.001, **p < 0.01, *p < 0.05, versus control. ^###^ *p <* 0.001, ^##^ *p <* 0.01, ^#^ *p <* 0.05, versus TGF‐β1.

Firstly, we used TGF‐β1 to stimulate mouse primary RTEC (Figure 4A) and mouse tubular epithelial TCMK‐1 cell line. The Cell Counting Kit‐8 (CCK‐8) assay was used to select the appropriate concentration by examining the cytotoxic effect of LBQ657 (0–200 μM) in TCMK‐1 cells. As shown in Figure S5A, there were no significant perturbations in cell viability when treated with LBQ657 at a concentration of 0, 1, 5, 10, and 20 μM, respectively. Furthermore, the protein expression of the fibrotic marker Fn, α‐SMA, and Col Iα1 was significantly increased in TCMK‐1 cells stimulated with TGF‐β1, while these fibrotic phenotypes were obviously mitigated by simultaneous treatment of 10 or 20 μM LBQ657 (Figure S5B). Moreover, LBQ657 significantly inhibited the elevated activity of NEP at a concentration of 10 μM in TCMK‐1 cells (Figures S5C and D). Therefore, we chose the 10 μM concentration of LBQ657 for the further in vitro experiments.

Next, we systematically evaluated the effects of LBQ657 on tubular injury and fibrosis in primary RTECs and TCMK‐1 cells. As delineated in Figures 4B and C, the mRNA levels of KIM‐1 and NGAL were significantly decreased when administered simultaneously with LBQ657 and TGF‐β1. In addition, TGF‐β1‐induced protein and mRNA expression of Fn, α‐SMA, and Col Iα1 was also significantly decreased by LBQ657 (Figures 4D–G and S6A, B). In general, these data indicated that inhibition of NEP could alleviate tubular injury and fibrotic phenotype in TGF‐β1‐induced tubular epithelial cells.

### 2.5 Ferroptotic phenotype of fibrotic kidneys was alleviated by NEP inhibition

**FIGURE 5:**
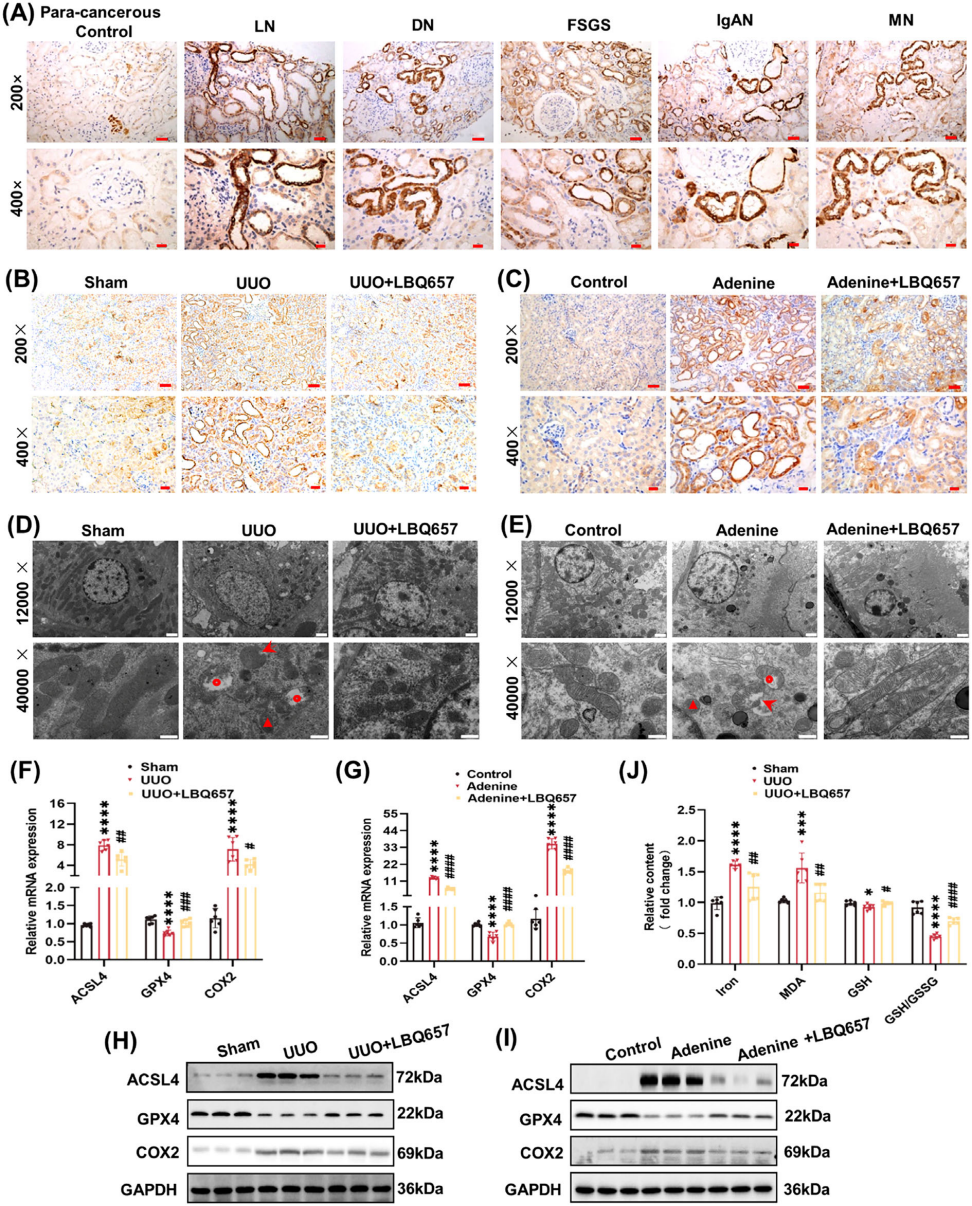
Ferroptosis in the fibrotic kidney was alleviated by inhibition of neprilysin. (A) Tubular ACSL4 was upregulated in kidney biopsies of CKD patients by immunochemistry staining (×200, scale bar = 50 μm; ×400, scale bar = 20 μm). (B and C) Immunochemistry staining of ACSL4 in mouse kidney sections (×200, scale bar = 50 μm; ×400, scale bar = 20 μm). LN, lupus nephritis; DN, diabetic nephropathy; FSGS, focal segmental glomerulosclerosis; IgAN, IgA nephropathy; MN, membranous nephropathy. (D and E) Transmission electron microscopy of mouse kidney sections (×12,000, scale bar = 1 μm; ×40,000, scale bar = 500 nm). Triangle: shrunken mitochondria; circle: absent mitochondrial ridge; arrow: rupture of the mitochondrial outer membrane. (F and G) RT‐PCR was performed to detect the mRNA expression of ACSL4, GPX4, and *Ptgs2* (COX2). (H and I) Western blot analysis was performed to detect protein expression of ACSL4, GPX4, and COX2. (J) Comparison of iron, MDA, GSH, and GSH/GSSG levels in the kidneys. ****p < 0.0001, ***p < 0.001, *p < 0.05, versus sham or control. ^####^ *p <* 0.0001, ^###^ *p <* 0.001, ^##^ *p <* 0.01, ^#^ *p <* 0.05, versus UUO or adenine.

ACSL4, a key enzyme in ferroptosis, participates in lipid peroxidation by converting free arachidonate to arachidonoyl‐CoA.^14^, ^24^ In particular, ACSL4 is a key contributor and predictive biomarker of ferroptosis.^14^, ^25^ In this study, ACSL4 was significantly increased in the kidneys of patients with CKD by immunohistochemical staining, its location was mainly in the renal tubular region regardless of the type of renal pathology (Figures 5A and S7A). Similar results were observed in the kidneys of UUO and adenine diet‐induced mice (Figures 5B, C and S7B, C). These results indicated that tubular ACSL4 expression was potentially involved in ferroptotic stress of kidney fibrosis.

Furthermore, the results of immunohistochemical staining indicated that increase in ACSL4 expression could be suppressed by inhibition of NEP in the kidneys of UUO and adenine diet‐induced mice (Figures 5B, C and S7B, C). Additionally, ferroptotic stress phenotype was detected in the kidneys of UUO and adenine diet‐induced mice by transmission electron microscopy, which was characterized by shrunken mitochondria, increased mitochondrial membrane density, reduced or absent mitochondrial ridge, and rupture of mitochondrial outer membrane, while nuclear size was normal without chromatin aggregation. These morphological alterations were effectively restored by inhibition of NEP with LBQ657 (Figures 5D and E).

To verify antiferroptotic effect of NEP inhibition, we further examined the transcriptional and translational levels of ferroptotic markers in the kidneys of UUO and adenine diet‐induced mice. In the present results, the level of glutathione peroxidase 4 (GPX4) mRNA and protein was downregulated while ACSL4 and *Ptgs2* (cyclooxygenase 2, COX2) were upregulated in kidneys of UUO and adenine diet‐induced mice. Importantly, LBQ657 restored the corresponding ferroptotic phenotype with antifibrotic effect (Figures 5F–I and S7D, E). It is well established that the increased iron, malondialdehyde (MDA) and the reduced glutathione (GSH) and glutathione/glutathione disulfide (GSH/GSSG) ratio are common markers of ferroptosis.^26^, ^27^ Similarly, we found that the higher level of iron, MDA, and the lower level of GSH and GSH/GSSG ratio in the kidneys of UUO mice, and these changes were reversed by inhibition of NEP (Figure 5J). Together, NEP inhibition protected against ferroptosis in the fibrotic kidneys.

### 2.6 NEP inhibition alleviated ferroptosis in TGF‐β1‐induced tubular epithelial cells

**FIGURE 6:**
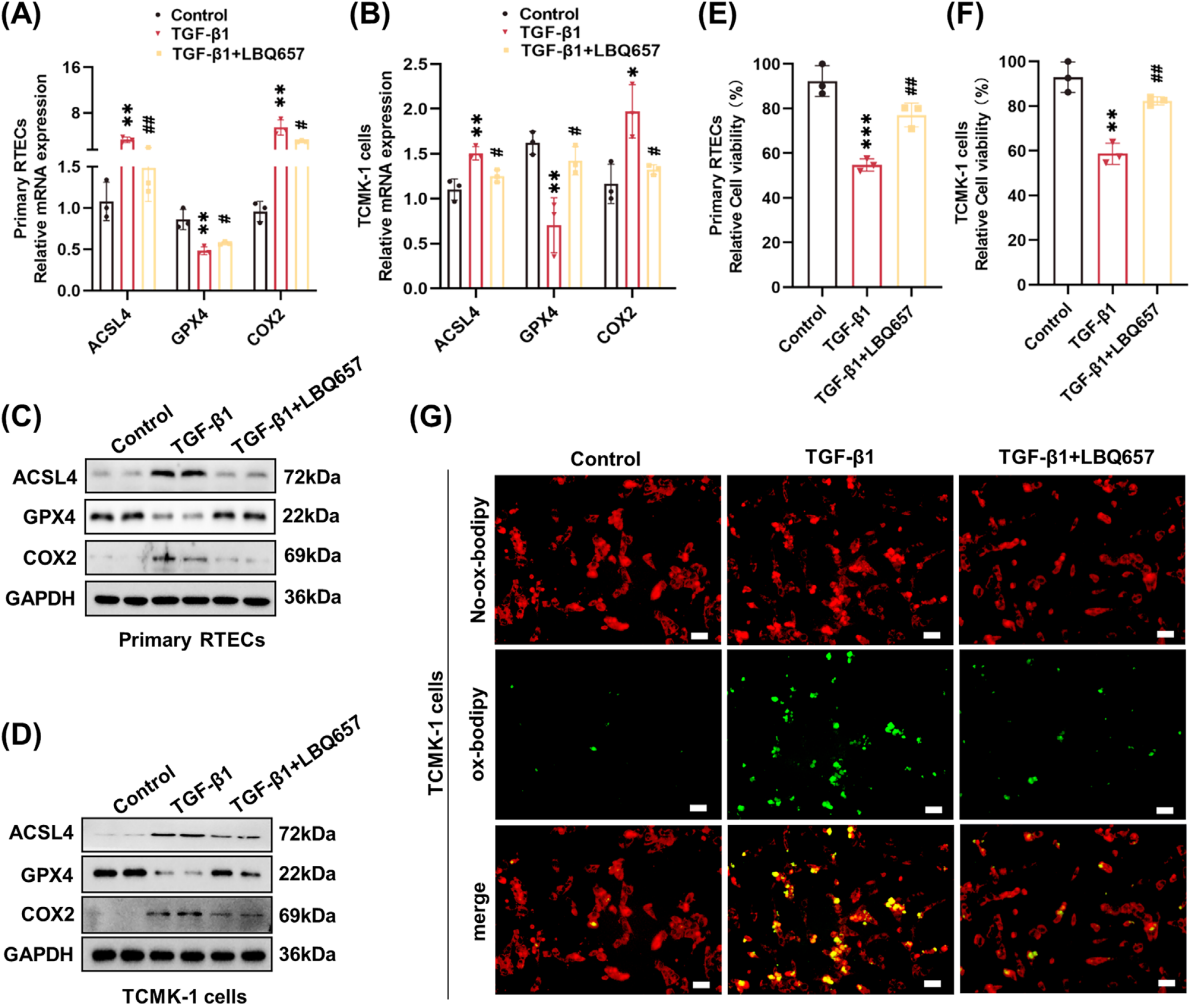
Pharmacological inhibition of neprilysin alleviated TGF‐β1‐induced ferroptosis in renal tubular cells. (A and B) RT‐PCR was performed to detect the mRNA level of ACSL4, GPX4, and *Ptgs2* (COX2) in primary RTECs and TCMK‐1 cells. (C and D) Western blot analysis was performed to detect ACSL4, GPX4, and COX2 protein expression in primary RTECs and TCMK‐1 cells. (E and F) Relative cell viability evaluated by the CCK‐8 assay. (G) The accumulation of lipid peroxidation in cells was analyzed by BODIPY C11 staining (×200, scale bar = 50 μm). ***p < 0.001, **p < 0.01, versus control. ^##^ *p <* 0.01, ^#^ *p <* 0.05, versus TGF‐β1.

The antiferroptosis effect of LBQ657 was further investigated in TGF‐β1‐induced primary RTECs and TCMK‐1 cells, respectively. As exhibited in Figures 6A–D and S8A, B, the downregulation of GPX4 and the upregulation of ACSL4 and COX2 were significantly attenuated by LBQ657. As cell viability is an indicator of cell death, we next examined the effect of LBQ657 on cell viability in TGF‐β1 stimulated RTECs. The results of the CCK‐8 assay indicated that tubular cell viabilities were markedly reduced by TGF‐β1, while they were significantly restored by LBQ657 administration in both primary RTECs and TCMK‐1 cells (Figures 6E and F). Furthermore, given that ROS interacts with cellular components and results in lipid peroxidation,^28^ we also evaluated the effect of LBQ657 on lipid ROS in TCMK‐1 cells. The BODIPY 581/591 C11 staining data showed that TGF‐β1 increased the level of oxidized lipid ROS in TCMK‐1 cells, present as an intensified green fluorescence, which were inhibited by LBQ657 (Figure 6G). In summary, these findings indicated that inhibition of NEP with LBQ657 exerted antiferroptotic effects in TGF‐β1‐induced RTECs.

### 2.7 Genetic knockdown of NEP improved fibrotic and ferroptotic phenotype in TGF‐β1‐induced tubular epithelial cells

**FIGURE 7:**
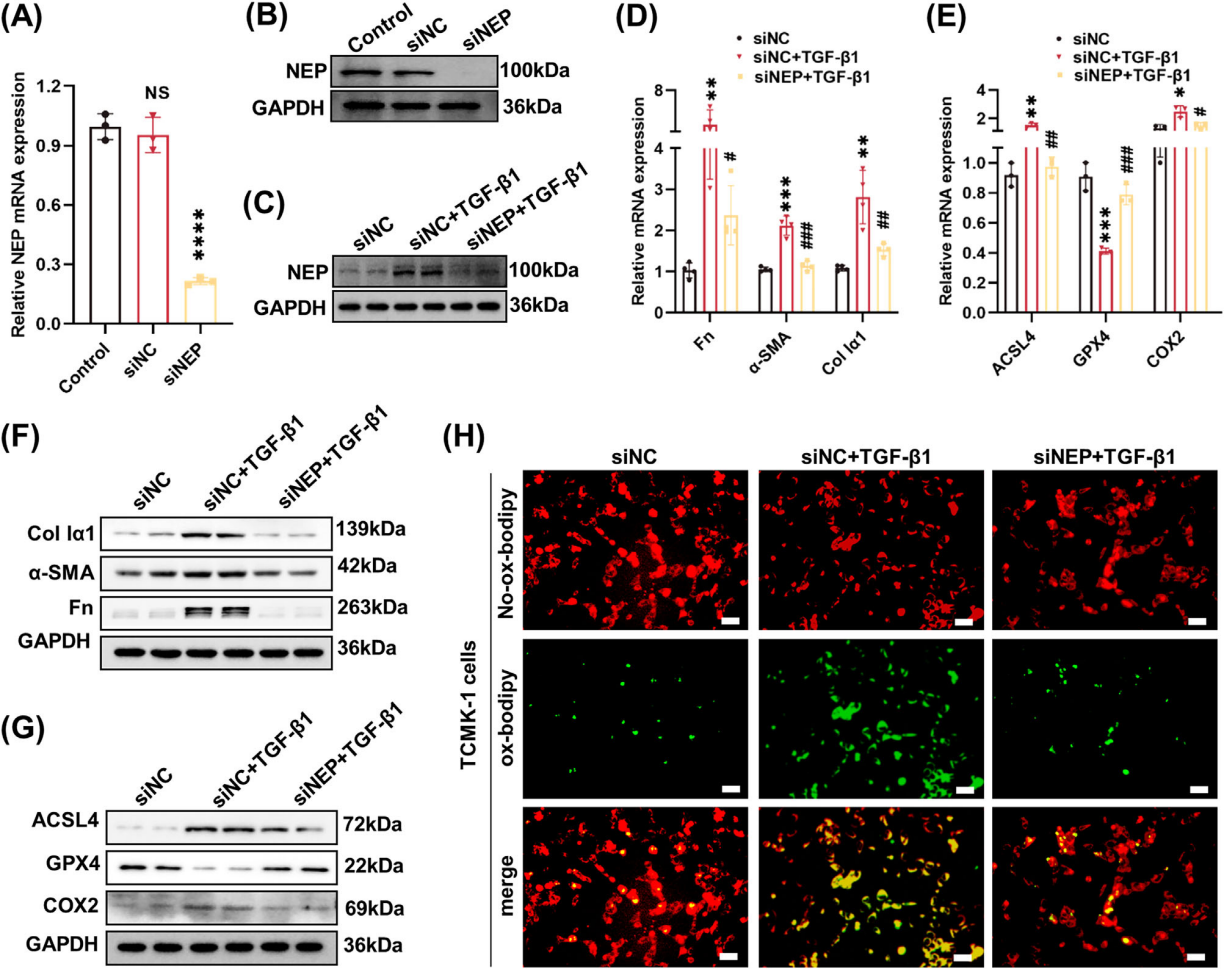
Genetic knockdown of neprilysin improved the fibrotic and ferroptotic phenotype in TGFβ1‐induced TCMK‐1 cells. (A and B) The knockdown efficiency of NEP siRNA (siNEP) in TCMK‐1 cells evaluated by RT‐PCR and Western blot analysis. (C) Representative Western blot images and quantitative analysis of NEP expression in TCMK‐1 cells. (D) RT‐PCR was performed to detect the mRNA level of Fn, α‐SMA, and Col Iα1. (E) RT‐PCR was performed to detect the mRNA level of ACSL4, GPX4, and *Ptgs2* (COX2). (F) Western blot analysis was performed to detect protein expression of Fn, α‐SMA, and Col Iα1. (G) Western blot analysis was performed to detect protein expression of ACSL4, GPX4, and COX2. (H) The accumulation of lipid peroxidation in TCMK‐1 cells was analyzed by BODIPY C11 staining (×200, scale bar = 50 μm). ^NS^ *p* > 0.05, versus control. ****p < 0.0001, ***p < 0.001, **p < 0.01, *p < 0.05, versus siNC. ^###^ *p <* 0.001, ^##^ *p <* 0.01, ^#^ *p <* 0.05, versus siNC+TGF‐β1. siNC, negative control.

TCMK‐1 cells were transfected with NEP siRNA (siNEP) to silence the expression of NEP gene and protein (Figure 7A, B and S9A). After TGF‐β1 stimulation, increased NEP expression was observed in TCMK‐1 cells, which was suppressed by NEP knockdown (Figures 7C and S9B). Furthermore, NEP knockdown attenuated TGF‐β1‐stimulated expression of the fibrotic protein Fn, α‐SMA, and Col Iα1 (Figures 7D, F and S9C). Meanwhile, the TGF‐β1‐induced ACSL4 and COX2 upregulation, and GPX4 downregulation were alleviated by NEP siRNA (Figures 7E, G and S9D). Additionally, NEP siRNA significantly improved tubular cell viability of TGF‐β1‐stimulated TCMK‐1 cells (Figure S9E). And the BODIPY 581/591 C11 staining data showed that the level of oxidized lipid ROS decreased markedly with NEP siRNA administration (Figure 7H). Collectively, these above‐mentioned results indicated that NEP knockdown by siRNA technology protected tubular injury against TGF‐β1‐induced fibrosis and ferroptosis.

### 2.8 NEP aggravated fibrotic phenotype by ACSL4‐mediated ferroptosis in TGF‐β1‐induced tubular epithelial cells

**FIGURE 8:**
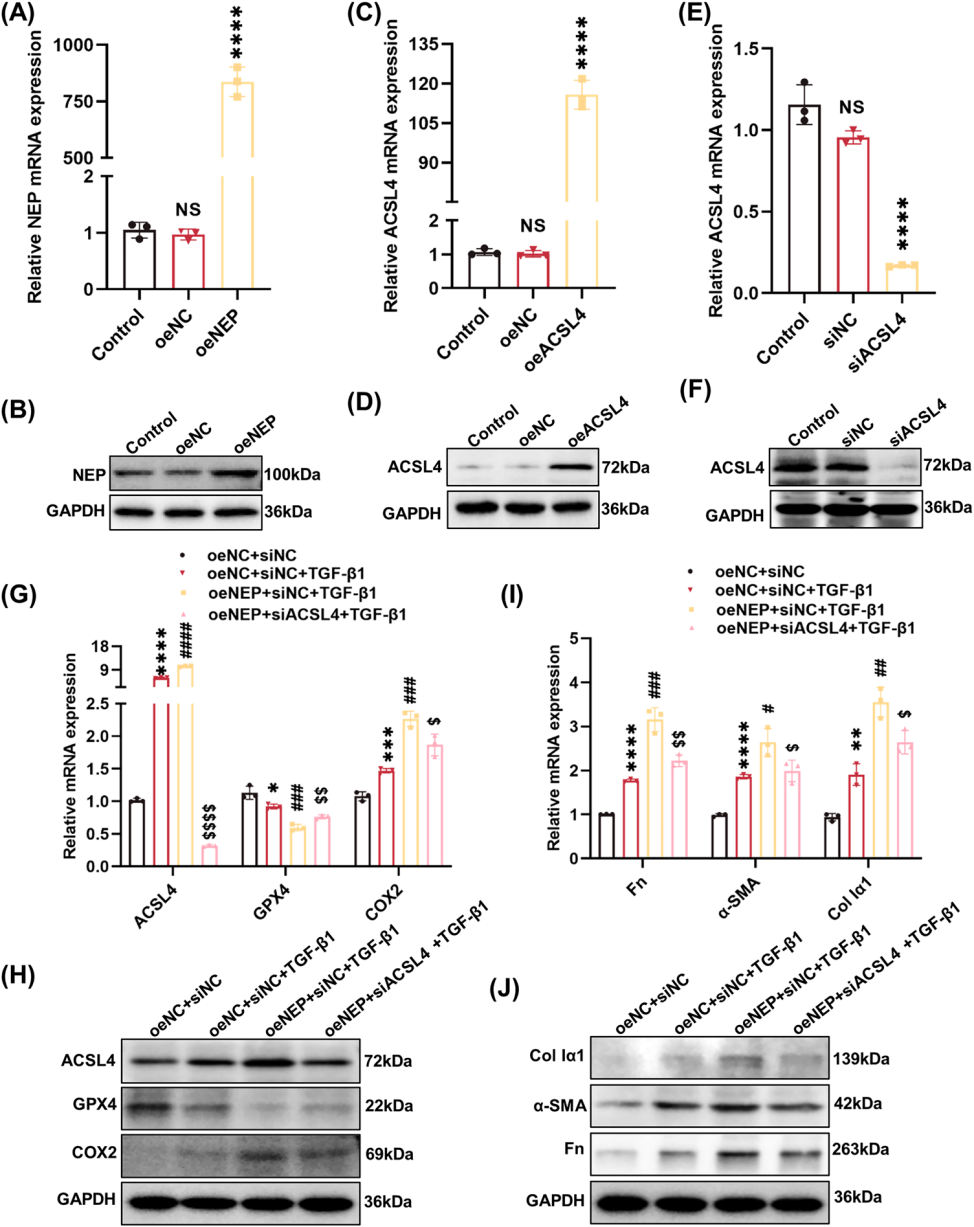
Neprilysin aggravated fibrosis by ACSL4‐mediated ferroptosis in TGF‐β1‐induced TCMK‐1 cells. (A and B) The efficiency of NEP overexpression in TCMK‐1 cells evaluated by RT‐PCR and Western blot analysis. (C and D) The overexpression efficiency of ACSL4 in TCMK‐1 cells evaluated by RT‐PCR and Western blot analysis. (E and F) The knockdown efficiency of ACSL4 siRNA in TCMK‐1 cells evaluated by RT‐PCR and Western blot analysis. (G) RT‐PCR was performed to detect the mRNA expression of ACSL4, GPX4, and *Ptgs2* (COX2). (H) Western blot analysis was performed to detect protein expression of ACSL4, GPX4, and COX2. (J) RT‐PCR was performed to detect the mRNA expression of Fn, α‐SMA, and Col Iα1. (I) Western blot analysis was performed to detect protein expression of Fn, α‐SMA, and Col Iα1. ****p < 0.0001, ***p < 0.001, **p < 0.01, *p < 0.05, versus oeNC or siNC or oeNC+siNC. ^####^ *p <* 0.0001, ^###^ *p <* 0.001, ^##^ *p <* 0.01, ^#^ *p <* 0.05, versus oeNC+siNC+TGF‐β1. ^$$$$^ *p <* 0.0001, ^$$^ *p <* 0.01, ^$^ *p <* 0.05, versus oeNEP+siNC+TGF‐β1. siNC, silencing negative control; siNEP, NEP siRNA; siACSL4, ACSL4 siRNA; oeNC, overexpression negative control; oeNEP, overexpression of NEP; oeACSL4, overexpression of ACSL4.

We investigated the effect of gene silencing and overexpression of NEP on ACSL4‐related ferroptosis and fibrotic phenotype in tubular cells. As shown in Figures 8A–F and S10A–C, the efficiency of gene silencing and overexpression of NEP or ACSL4 was verified by RT‐PCR analysis and Western blot analysis in TCMK‐1 cells. Then, NEP overexpression resulted in a greater elevation of ACSL4, COX2, and a greater reduction of GPX4 in TGF‐β1‐stimulated TCMK‐1 cells, which indicated that NEP aggravated tubular cell ferroptosis. Importantly, ACSL4 knockdown attenuated NEP overexpression worsening of tubular cell ferroptosis in TGF‐β1‐stimulated TCMK‐1 cells (Figures 8G, H and S10D). Consistently, upregulation of fibrotic markers (Fn, α‐SMA, and Col Iα1) was also increased by overexpression of NEP, while these corresponding changes were also diminished by silencing of ACSL4 (Figures 8I, J and S10E). These results suggested that NEP overexpression aggravated the fibrotic phenotype through ACSL4‐mediated ferroptosis in TGF‐β1‐stimulated tubular cells.

Furthermore, the NEP siRNA transfection resulted in decreased expression of ACSL4 and COX2, and increased expression of GPX4 in TGF‐β1‐stimulated TCMK‐1 cells, where antiferroptotic effect of NEP knockdown was diminished by overexpression of ACSL4 with the recombinant plasmid (Figures S11A and B). Meanwhile, the fibrotic markers (Fn, α‐SMA, and Col Iα1) were down‐regulated by transfection of NEP siRNA in TGF‐β1‐stimulated TCMK‐1 cells, while all the corresponding changes were re‐upregulated by overexpression of ACSL4 (Figures S11C and D). And NEP inhibition with LBQ657 exhibited a similar effect to NEP knockdown (Figures S11E–H). These findings further demonstrated that NEP inhibition alleviated fibrotic phenotype through ACSL4‐mediated ferroptosis in TGF‐β1‐induced tubular cells.

## 3 DISCUSSION

In this study, NEP expression was markedly increased in tubular epithelial cells of CKD patients and two kinds of CKD mouse models, where inhibition of NEP could improve tubular ferroptosis and kidney fibrosis. The results identified the renoprotective effect of NEP inhibition in CKD mice and TGF‐β1‐stimulated tubular epithelial cells. Mechanically, NEP overexpression aggravated the ferroptotic and fibrotic phenotype in TGF‐β1‐induced tubular cells, while this phenomenon was restored by ACSL4 knockdown. NEP knockdown attenuated tubular cell ferroptosis, which was exacerbated by ACSL4 overexpression. Together, our data provided a new insight into the relationship between NEP and tubular ferroptosis, and revealed that inhibition of NEP exerted a promising therapeutic against kidney fibrosis.

NEP accounts for approximately 4% of all membrane proteins in the kidney, where NEP is expressed mainly in the brush border membrane of the proximal renal tubule.^29^ Inhibition of NEP has the potential to exert a renoprotective effect in heart failure patients and UUO mice.^11^, ^30^ However, the mechanism of tubular NEP in kidney fibrosis remains obscure. Similar to the previous study, we found that systemic administration of the NEP inhibitor *Sacubitrilat* (LBQ657) could improve tubular injury, restore renal function, and retard kidney fibrosis in mouse models. The antifibrotic effect of NEP inhibition by LBQ657 and gene knockdown was further confirmed in TGF‐β1‐stimulated tubular epithelial cells. Furthermore, tubular cell ferroptosis was explored in the fibrotic kidneys of CKD patients and mice, which was impeded by LBQ657 treatment in both UUO and adenine diet‐induced mice, as well as in TGFβ1‐stimulated RTECs. Thus, for the first time, we speculated that NEP participated in kidney fibrosis via tubular cell ferroptosis.

Recently, an expanding body of evidence demonstrated the activation of ferroptosis in CKD, and reported that ferroptosis inhibition could exert antifibrotic effects in the kidney. For example, Fer‐1, a specific ferroptosis inhibitor, could alleviate graphene quantum dots‐induced fibrosis in the kidney.^31^ In fibrotic kidneys after I/RI or UUO treated with ferroptosis inhibitor, the kidney injury, interstitial fibrosis and inflammatory cell accumulation were significantly diminished.^15^ In the 5/6 nephrectomy rat model, tubular injury markers and fibrosis were detected, and the changes were exacerbated by ferroptosis inducer, and reversed by ferroptosis inhibitor.^16^ Although ferroptosis inhibition was shown to alleviate renal injury, inflammation, and fibrosis, how NEP regulates tubular ferroptosis in kidney fibrosis remains unknown.

The hallmark of ferroptosis is lipid peroxidation. ACSL4 is a pivotal enzyme involved in lipid peroxidation and has been considered a critical positive regulator and biomarker of ferroptosis.^25^ ACSL4 silencing reduced ferroptosis sensitivity, while its overexpression aggravated the cellular lipid composition and evoked ferroptosis.^14^, ^25^ In the current study, we found that ACSL4 was upregulated in the kidneys of CKD patients and mice, and the location of expression was mainly in the renal tubular region. Although both NEP and ACSL4 were found to be expressed in tubular cells, there has so far been no evidence regarding their relationship. In this study, NEP siRNA transfection decreased fibrotic protein expression in TGF‐β1‐induced RTECs. Furthermore, NEP overexpression amplified the profibrotic effect of TGF‐β1‐induced RTECs, while ACSL4 silencing could partially counteract the effect of NEP overexpression. Once ACSL4 was overexpressed, antifibrotic effect of NEP inhibition was weakened. Taken together, our data indicated that NEP‐regulating tubular cell ferroptosis played an important role in kidney fibrosis. Theoretically, ferroptotic tubular epithelial cells secrete high mobility group box 1 (Figure S12), which may promote inflammation and transdifferentiation of RTECs into myofibroblasts, and lead to fibrosis.^32^, ^33^

However, the hyperfine interaction between NEP and ACSL4‐mediated ferroptosis remains obscure. Functionally, NEP may indirectly lead to alteration of ACSL4 expression by affecting some peptides, and the specific mechanism warrants exploration in the future. Furthermore, the effect of NEP overexpression on tubular fibrotic phenotype was partially alleviated by the deletion of the ACSL4 gene, and, in parallel, the effect of NEP knockdown on fibrosis was also partially aggravated by ACSL4 overexpression. These results indicated that additional mechanisms might exist that have not yet been elucidated. In summary, a novel aspect of NEP for the first time was explored in kidney fibrosis through ACSL4‐meidated tubular epithelial cell ferroptosis. Furthermore, our data further indicated that NEP inhibition exerted a promising therapeutic against fibrotic kidney diseases.

## 4 MATERIALS AND METHODS

### 4.1 Chemicals and antibodies

Sacubitrilat (LBQ657, 149709‐44‐4, purity 99.8%) was purchased from Bohui Medical Technology Co., Ltd (Nanjing, China). TGF‐β1 (Z03411‐50, purity>95%) was from GenScript Probio (Nanjing, China). Primary antibodies are shown in Table S1.

### 4.2 Human biopsy samples

All human renal biopsy tissues were collected from CKD patients in West China Hospital of Sichuan University. The Para‐cancerous renal tissues were used as controls. Written informed consent was provided by each patient or their guardians prior to participating in the study. And it was approved by the ethics committee of West China Hospital of Sichuan University (No. 2016−273) and was in compliance with the Declaration of Helsinki.

### 4.3 Animal experiments

Male C57BL/6J mice (8–10 weeks, weight 20−25 g) were purchased from GemPharmatech Co., LTD (Nanjing, China). Mice were housed in standardized conditions (12 h/12 h dark/light cycle) at 25°C. All mice accessed to diet and water ad libitum and adapted to this environment for 1 week before further research.

In the UUO model, the mice were randomly divided into three groups: Sham (*n* = 6), UUO (*n* = 6), and UUO + LBQ657 (*n* = 6). The UUO model was established as previously described.^34^ Sham mice were operated in a similar manner, but without ureteral ligation. The UUO + LBQ657 group received LBQ657 (30 mg/kg/day) by oral gavage after ligating the left ureter (Figure S13A), while the sham and UUO groups received the same volume of phosphate buffer saline by oral gavage for 7 consecutive days. In the adenine model, mice were randomly assigned to three groups: control (*n* = 6), adenine (*n* = 6), adenine + LBQ657 (n = 6). The adenine model was established by feeding mice with an adenine‐containing diet (0.2% adenine) consecutively for 14 days, and mice from the control group received normal adenine‐free feed according to the same feeding schedule. LBQ657 (30 mg/kg/day) administration was performed with an oral gavage as mentioned above simultaneously (Figure S13B). Meanwhile, the control and adenine groups received same‐volume phosphate buffer saline by oral gavage.

At the end of the experiment, both batches of mice were sacrificed, respectively, by intraperitoneal injection of sodium pentobarbital (50 mg/kg), the serum and kidney samples were collected and stored at −80°C. All experimental protocols and animal procedures were approved by the Animal Care and Use Ethics Committee of Sichuan University (No. 20220602002).

### 4.4 Serum biochemistry assays

Serum was isolated by centrifugation (1811×*g*, 20 min) and Scr and BUN levels were measured using an automatic biochemical analyzer (BS‐240; Mindrary, Shenzhen, China).

### 4.5 Histological examination

Kidney tissues were fixed in 10% neutral‐buffered formalin, dehydrated with gradient alcohol solutions, and embedded in paraffin. Tissue sections were cut at 4 μm thickness, and then subjected to HE and Masson's trichrome staining after being deparaffinized and rehydrated. The sections were imaged using an AxioCamHRc digital camera (Carl Zeiss, Jena, Germany) for morphologic analysis. The tubular injury score was calculated by two independent pathologists under double‐blind conditions and performed in accordance with previously described methods.^35^

### 4.6 Immunohistochemistry

Immunohistochemical staining was performed according to previous reports.^36^ Primary antibodies against NEP (1:200, 18008‐1‐AP; Proteintech, USA), α‐SMA (1:100, ET1607‐43; Huabio, China), and ACSL4 (1:200, 81196‐1‐RR; Proteintech) were used in the study. Images were acquired using an AxioCamHRc digital camera (Carl Zeiss).

### 4.7 Transmission electron microscopy

Kidney tissues were sequentially fixed with 3% glutaraldehyde and 1% osmium tetroxide, then dehydrated with gradient acetone and coated with Epon812. Tissue sections with a thickness of 60–90 nm were obtained using a Leica EM UC7 ultramicrotome, and stained with uranium acetate and lead citrate successively. Images were captured with a JEM‐1400‐FLASH transmission electron microscope.

### 4.8 Western blot analysis

Kidney tissues or mouse RTECs were homogenized with cold 1×SDS‐PAGE loading buffer (Biyotime, Shanghai, China). After centrifugation (3579×*g*) at 4°C for 15 min, the supernatants were collected and boiled at 100°C for 10 min. Protein samples were separated on 10−15% SDS‐polyacrylamide gels and then transferred onto PVDF membranes (Bio‐Rad, Hercules, CA, USA). After blocking, the membranes were then sequentially incubated with corresponding primary and secondary antibodies. The protein bands were visualized by using the hypersensitive chemiluminescent ECL reagents (Lamrol bio, Chengdu, China) and a Clinx ChemiScope 5800 imaging system. The band densitometry was quantified using the NIH ImageJ version 1.51 program.

### 4.9 Quantitative real‐time PCR analysis

Total RNA was extracted from kidney tissues or mouse kidney tubular cells using a total RNA isolation kit (TP‐01121; Foregene, Chengdu, China). RNA concentration was determined by the Nano‐500™ Micro‐Spectrophotometer (Allsheng, hangzhou, China). Reverse transcription was carried out using HiScript III RT SuperMix for qPCR (Vazyme, Nanjing, China). Quantitative real‐time PCR (RT‐PCR) was performed with iTaq™ Universal SYBR Green Supermix (Bio‐Rad) on a PCR system (CFX Connect; Bio‐Rad). Data were analyzed using the comparative 2^−ΔΔCT^ method, and GAPDH was selected as an internal reference. Primer sequences are shown in Table S2.

### 4.10 RNA‐Seq transcriptomic assay

Three independent kidney samples were selected from each group (Sham and UUO group) for sequencing. Total RNA was isolated with Trizol reagent (Invitrogen, Carlsbad, CA, USA) as previously described.^37^ Library construction and sequencing were performed by Majorbio Bio‐tech Ltd (Shanghai, China). Advanced Heatmap Plots were created using the OmicStudio tools at https://www.omicstudio.cn.

### 4.11 Isolation and culture of primary tubular epithelia cells

Male C57BL/6J mice, aged 2−3 weeks, were used for the primary RTECs isolation procedures. Concisely, after anesthesia, both kidneys of the mice were harvested. After removing the capsule and the renal pedicle, the renal cortex was divided into tiny pieces (<1 mm) and incubated with 1 mg/mL collagenase Type I (17100‐017; Gibco, MA, USA) in a 37°C shaker at 22×*g* for 40 min. Subsequently, the samples were passed through sieves of descending pore sizes (100, 70, and 40 μm). The filtrate containing dissociated tubular epithelial cells was then centrifuged at 275×*g* for 10 min at 4°C. The supernatant was removed and the samples were incubated with red blood cell lysis buffer (R1010; Solarbio, Beijing, China) for 3 min on ice. Subsequently, 8 mL of PBS was added and centrifuged at 1000 rpm for 5 min at 4°C. Finally, the supernatants were removed and primary RTECs were resuspended and cultured in RPMI1640 (HyClone, Logan, UT, USA) with 10% fetal bovine serum (FBS) (35‐081‐CV; Corning, NY, USA), 1×insulin transferrin selenium additive (abs9462; Absin, Shanghai, China), 1% penicillin/streptomycin (S110JV; BasalMedia, Shanghai, China), and 20 ng/mL epidermal growth factor (RP‐10914; ThermoFisher, Waltham, MA, USA) in 5% CO_2_ at 37°C. After one subculture, cells were first starved in DMEM (CTCC‐002‐008; Meisen CTCC, Zhejiang, China) containing 0.5% FBS for 24 h, and then exposed to TGF‐β1 (20 ng/mL) for another 24 h with or without LBQ657 treatment.

### 4.12 TCMK‐1 cell culture and treatments

Mouse kidney tubular epithelium cells (TCMK‐1) (ATCC^®^ CCL‐139™; Beijing bnbio Co. Ltd, Beijing, China) were cultured in DMEM supplemented with 10% FBS in 5% CO_2_ at 37 °C. when in the logarithmic growth phase, cells were firstly starved for 24 h in DMEM containing 0.5% FBS, and then exposed to TGF‐β1 (20 ng/mL) for another 24 h with or without LBQ657 treatment in DMEM containing 10% FBS. Gene silencing and overexpression experiments were performed in the serum starvation stage.

### 4.13 Transfection of small interfering RNA and plasmid

Small interfering RNAs (siRNA) were purchased from GenePharma (Shanghai, China). The sequences of siRNAs used for transfection are shown in Table S3. Commercialized recombinant plasmids for NEP (Miaoling; P40790) and ACSL4 (Miaoling; P32185) and scrambled plasmids (Miaoling; P8196) were purchased from the Miaoling Plasmid Platform (miaolingbio.com). TCMK‐1 cells were transfected with siRNA or plasmid using Lipofectamine^®^ 2000 transfection reagent (11668‐019; Invitrogen) according to the manufacturer's instructions. The method for cotransfection of plasmid DNA and siRNA was constructed following a method as for plasmid DNA alone, with some adaptations. First, the plasmid DNA solution (1 μg of plasmid DNA in 250 μL serum‐free antibiotic‐free RIPM1640 medium) and the transfection reagent solution (10 μL Lipo2000™ in 250 μL serum‐free antibiotic‐free RIPM1640 medium) were prepared separately and incubated at room temperature for 5 min. The plasmid DNA solution and transfection reagent solution were then gently mixed and sat at room temperature for 20 min. Logarithmically growing cells in six‐well plates were selected and the medium was replaced by 1 mL of fresh serum‐free antibiotic‐free RPMI1640, and the transfection mixture was added to the cells dropwise. After 4–6 h of transfection, the culture medium was replaced with DMEM containing 0.5% FBS. Approximately 24 h after transfection, the media was changed to DMEM containing 10% FBS, and stimulation of TGF‐β1 (20 ng/mL) was performed whenever necessary.

### 4.14 Cell viability assay

A CCK‐8 assay (APExBIO, Houston, TX, USA) was used to examine cell viability. TCMK‐1 cells of the logarithmic growth phase were seeded into 96‐well culture plates at a density of 5000 cells/well. After specific stimulations, the culture medium was replaced by fresh DMEM containing 10% FBS and 10% CCK‐8 reagent, then incubated in the dark for at least 30 min at 37°C. Finally, the absorbance was determined by a microplate reader (Synergy Mx, Biotek, Vermont, USA) at 450 nm.

### 4.15 Lipid ROS measurement

The generation of lipid ROS was evaluated with a BODIPY 581/591 C11 probe (D3861; Invitrogen). After the preintervention measures were completed, the cell culture medium was replaced by fresh DMEM containing 10% FBS and 5 μM BODIPY 581/591 C11 solution. The cells were then incubated in an incubator for 30 min (dark, 5% CO2, 37°C). After incubation, the medium was replaced with fresh DMEM containing 10% FBS, and images were captured by an AxioCamHRc digital camera (Carl Zeiss).

### 4.16 Iron quantification

Renal iron content was measured using the Iron Colorimetric Assay Kit (ab83366; Abcam, Shanghai, China). Renal tissue (10 mg) was homogenized in 100 μL of the Iron Assay Buffer using a homogenizer (Servicebio, Wuhan, China) at 4°C, then centrifuged at 16,000×*g* for 10 min. The supernatant was collected for measurement of iron concentrations according to the commercialized assay procedure.

### 4.17 MDA content assay

Renal MDA levels were determined by a Lipid Peroxidation MDA Assay Kit (S0131S; Beyotime, Shanghai, China). Renal tissue (10 mg) was homogenized in 100 μL PBS using a homogenizer (Servicebio) at 4°C, and the sample solution was clarified by centrifugation (10,000×*g*) at 4°C for 10 min. The MDA content in the renal sample was determined strictly in accordance with the kit instructions.

### 4.18 Measurement of GSH and GSSG

The levels of renal GSH and GSSG were determined using a GSH and GSSG Assay Kit (S0053; Beyotime). Renal tissue (10 mg) was homogenized in 100 μL of protein removal reagent using a homogenizer (Servicebio) at 4°C. After standing for 5 min at 4°C, the sample was centrifuged at 2753×*g* for 10 min. The supernatant was extracted for following measurement of the total GSH content according to the kit instructions. Sample preparations for the GSSG measurement were performed as follows. Initially, an appropriate amount of the supernatant above was thoroughly mixed with the diluted GSH clearance auxiliary solution (5:1), then a certain amount of GSH scavenging solution was added (4%), thoroughly mixed, and incubated for 60 min at 25°C. Following the aforementioned treatments, the GSSG content of the renal samples were detected according to the manufacturer's guidelines.

### 4.19 NEP activity and BK content assay

NEP activity was assessed using the mouse NEP assay kit (JM‐11630M1; Jingmei, Jiangsu, China), and BK content was assessed using the mouse BK assay kit (JM‐02833M1; Jingmei). The experimental processes were carried out in strict accordance with the manufacturer's instructions.

### 4.20 Statistical analysis

All experiments were repeated at least three times. Quantitative experimental data were represented as mean ± SD. Statistical significance was determined using GraphPad Prism (Version 9; GraphPad Software, San Diego, CA, USA). Statistical differences were compared using a one‐way analysis of variance followed by a Tukey's post‐hoc test or by using a two‐tailed *t*‐test. Individual data points related to bar graphs were shown where applicable. Statistically significant was considered as *p*  <  0.05.

## AUTHOR CONTRIBUTION

L. Ma, P. Fu, W. Lai, and R. Huang designed the experiments. W. Lai, R. Huang, B. Wang, M. Shi, F. Guo, L. Li, Q. Ren, and S. Tao performed the experiments. W. Lai, R. Huang, and L. Ma analyzed the data. W. Lai, R. Huang, and L. Ma wrote the draft of the manuscript and edited it. All authors have read and approved the submission.

## CONFLICT OF INTEREST STATEMENT

The authors declare no conflict of interest.

## ETHICS STATEMENT

The study was approved by the ethics committee of West China Hospital of Sichuan University (No. 2016−273) and the Animal Care and Use Ethics Committee of Sichuan University (No. 20220602002). Written informed consent was provided by each patient or their guardians prior to participating in the study.

## Supporting information

Supporting Information

## Data Availability

All data supporting the findings of this study are available from the corresponding authors upon reasonable request.
