# Supplementary material for: Novel aspect of neprilysin in kidney fibrosis via ACSL4‐mediated ferroptosis of tubular epithelial cells
Source: MedComm (2020). 2023 Jul 14;4(4):e330. doi: 10.1002/mco2.330 (PMC10349188; doi:10.1002/mco2.330)
Supplement: Supplementary file 1 — Supporting Information [file MCO2-4-e330-s001.docx]

**Novel aspect of neprilysin in kidney fibrosis via ACSL4-mediated ferroptosis of tubular epithelial cells**

Weijing Lai^1,2#^, Rongshuang Huang^1#^, Bo Wang^1^, Min Shi^1^, Fan Guo^1^, Lingzhi Li^1^, Qian Ren^1^, Sibei Tao^1^, Ping Fu^1^*, Liang Ma^1^*

^1^ Department of Nephrology, Kidney Research Institute, West China Hospital of Sichuan University, Chengdu 610041, China.

^2^ Department of Nephrology, Clinical Medical College and The First Affiliated Hospital of Chengdu Medical College, Chengdu 610500, China.

^#^These authors contributed equally: Weijing Lai, Rongshuang Huang

*Correspondence: Ping Fu ([fupinghx@scu.edu.cn](mailto:fupinghx@scu.edu.cn)), Liang Ma ([liang_m@scu.edu.cn](mailto:liang_m@scu.edu.cn))


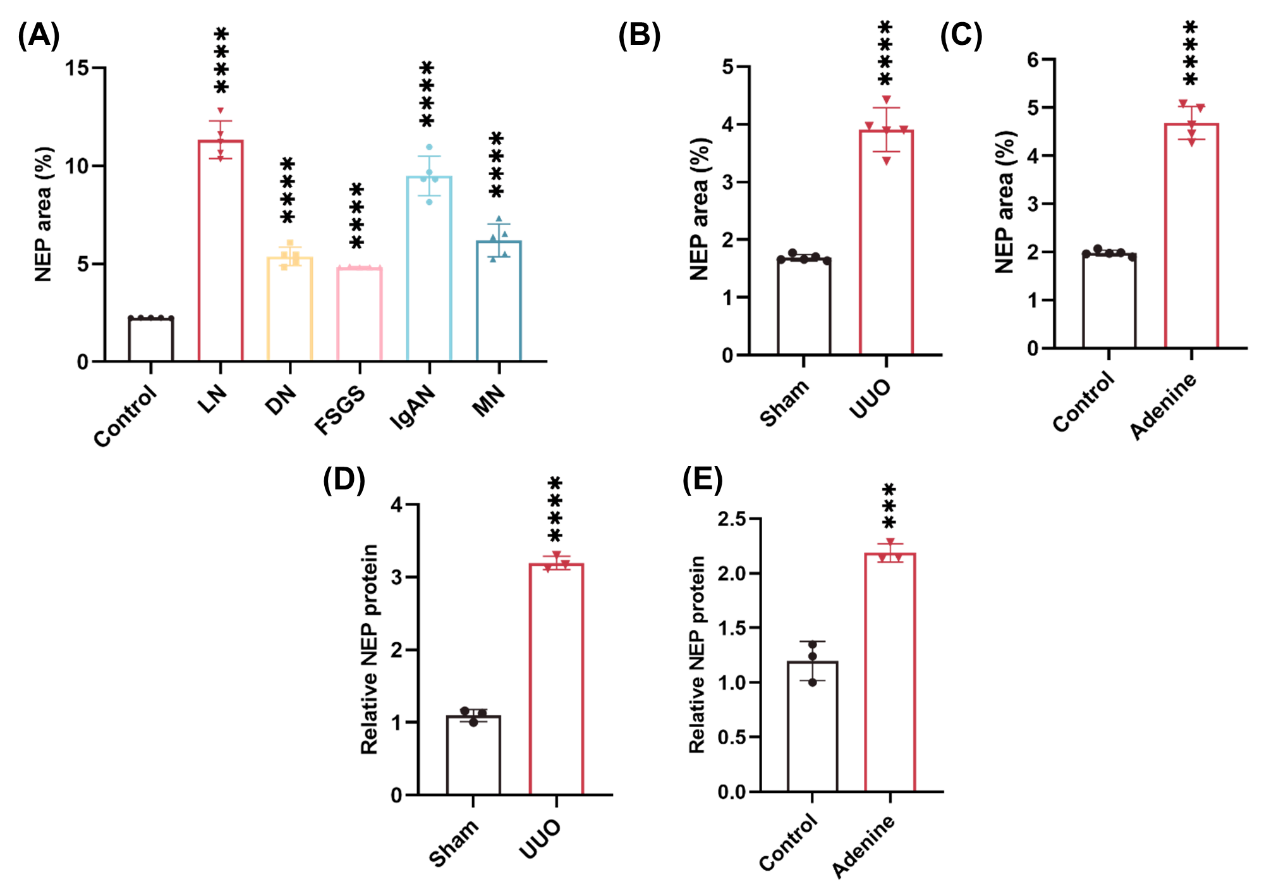
**FIGURE S1** **Renal neprilysin (NEP) was abnormally elevated in chronic kidney disease patients and mice.** (A) Percentage of NEP-positive area in human kidney sections. LN, lupus nephritis; DN, diabetic Nephropathy; FSGS, focal segmental glomerulosclerosis; IgAN, IgA nephropathy; MN, membranous nephropathy. (B,C) Percentage of NEP-positive area in mouse kidney sections. (D,E) Semi-quantification of western blot was performed to detect protein expression of NEP. *****P <* 0.0001, ****P <* 0.001, versus Sham or Control.

**
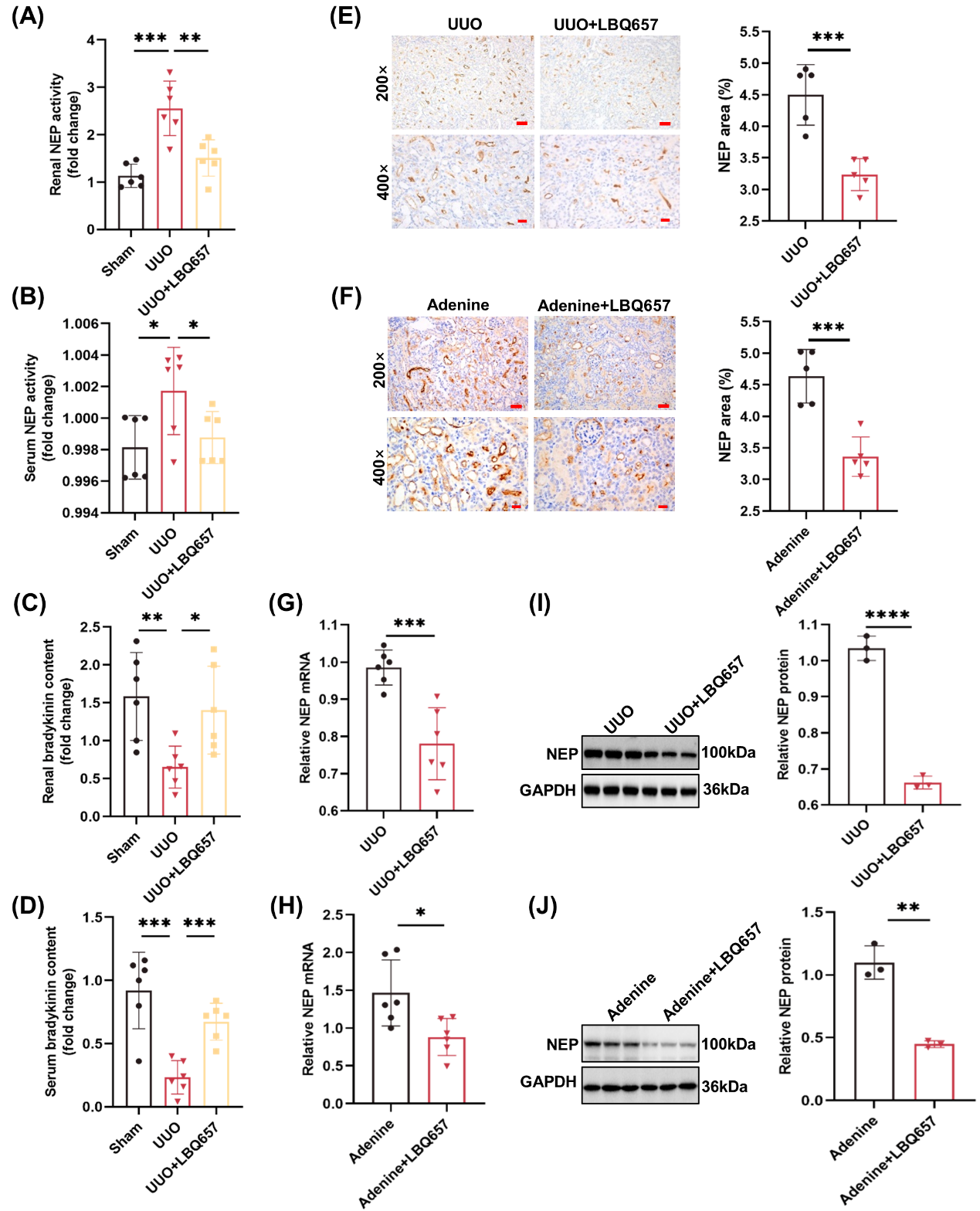
FIGURE S2** ***Sacubitrilat* (LBQ657) inhibited neprilysin (NEP) expression and activity in UUO and adenine diet-induced mice.** (A,B) NEP activity assayed by mouse NEP assay kit. (C,D) Bradykinin (BK) content was assayed by mouse BK assay kit. (E,F) Immunochemistry staining of NEP in mouse kidney sections (×200, scale bar = 50 μm; ×400, scale bar = 20 μm). (G,H) RT-PCR was performed to detect the expression of NEP mRNA. (I,J) Western blot analysis was performed to detect the expression of NEP protein. *****P <* 0.0001, ****P <* 0.001, ***P <* 0.01, **P <* 0.05.


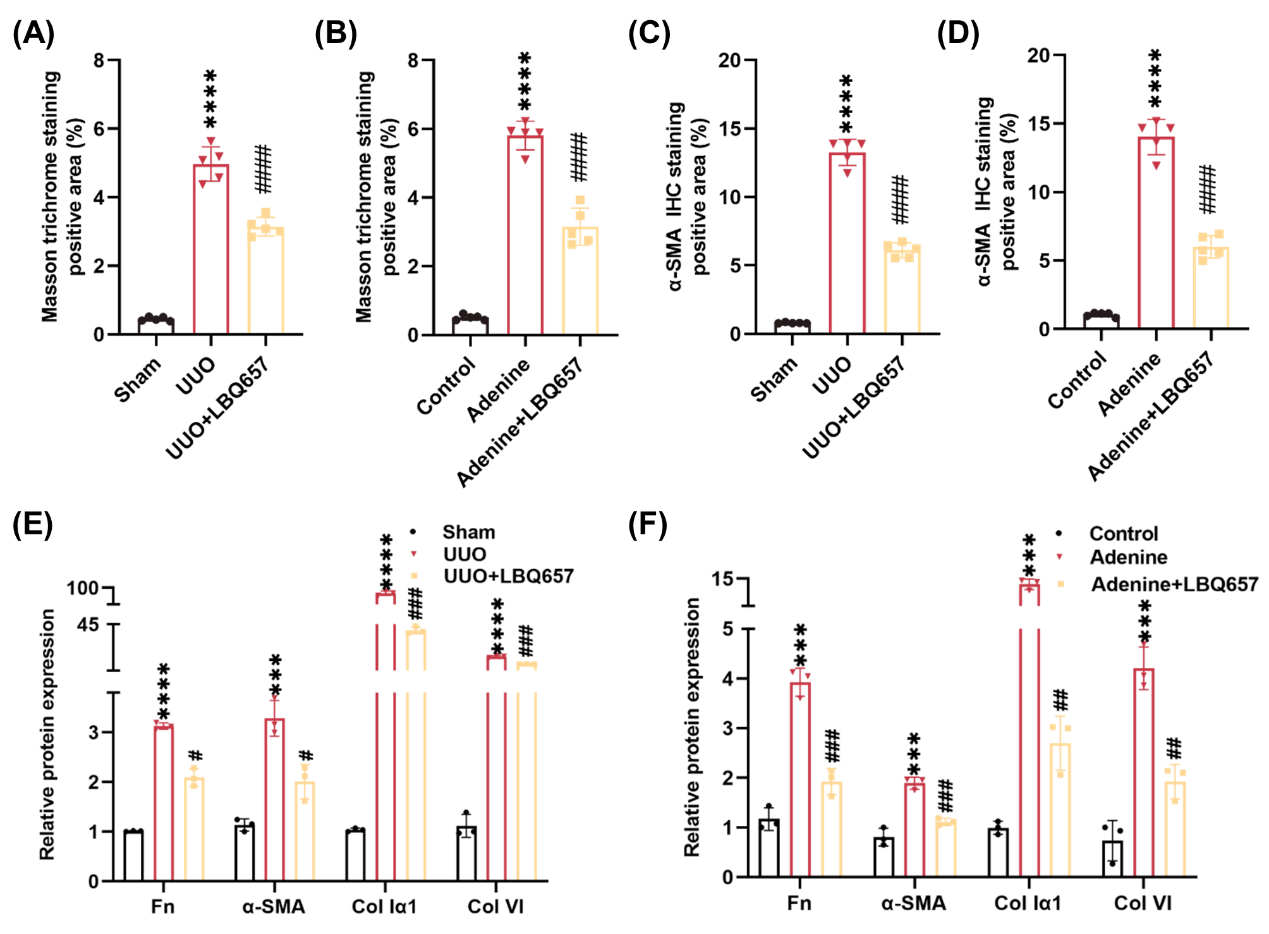


**FIGURE S3 Pharmacological inhibition of neprilysin (NEP) alleviated kidney fibrosis in mice induced by UUO and adenine diet.** (A,B) Percentage of Masson’s trichrome staining-positive area in mouse kidney sections. (C,D) Percentage of α-SMA-positive area in mouse kidney sections. (E,F) Semi-quantification of western blot was performed to detect protein expression of Fn, α-SMA, Col Iα1 and Col VI. *****P <* 0.0001, ****P <* 0.001, versus Sham or Control. ^####^*P <* 0.0001, ^###^*P <* 0.001, ^##^*P <* 0.01, ^#^*P <* 0.05, versus UUO or Adenine.

**
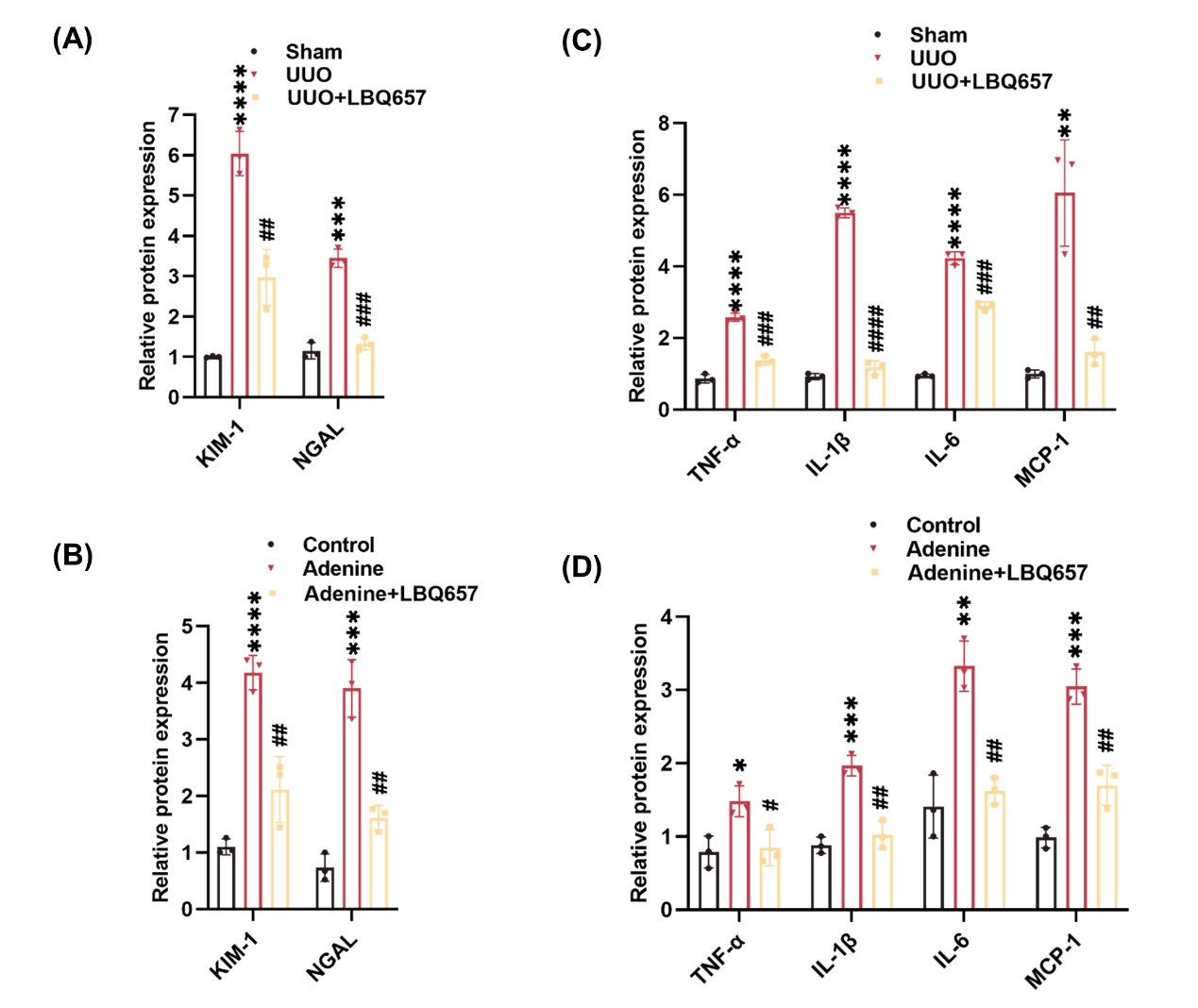
**

**FIGURE S4** **Neprilysin inhibition alleviated tubular injury and kidney inflammation in mice induced by UUO and adenine diet.** (A,B) Semi-quantification of Western blot was performed to detect protein expression of KIM-1 and NGAL. (C,D) Semi-quantification of Western blot was performed to detect the protein expression of MCP-1, IL-6, IL-1β and TNF-α. *****P<*0.0001, ****P<*0.001, ***P<*0.01, **P<*0.05, versus Sham or Control. ^####^*P<*0.0001, ^###^*P<*0.001, ^##^*P<*0.01, ^#^*P<*0.05, versus UUO or Adenine.


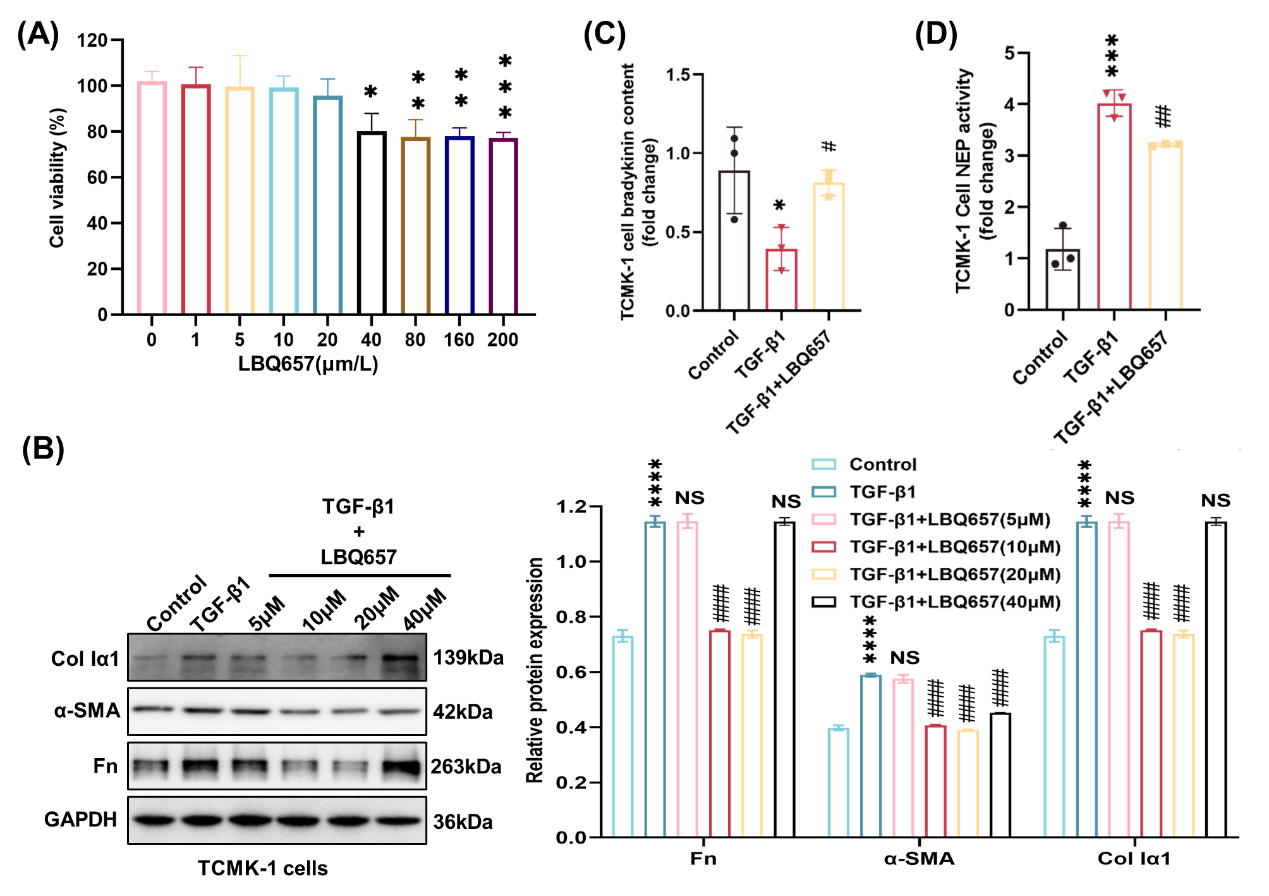


**FIGURE S5** **The reasonable option of *Sacubitrilat* (LBQ657) concentration.** (A) Cell viability of TCMK-1 cells treated with different concentrations of LBQ657. (B) Western blot analysis was performed to detect protein expression of Fn, α-SMA, and Col Iα1. (C) Neprilysin (NEP) activity was assayed by a mouse NEP assay kit. (D) Bradykinin (BK) content was assayed by mouse BK assay kit. *****P <* 0.0001, ****P <* 0.001, ***P <* 0.01, **P <* 0.05 versus Control (LBQ657 0 μM); ^####^*P*<0.0001, ^##^*P*<0.01, ^#^*P*<0.05, ^NS^*P* > 0.05, versus TGF-β1.

**
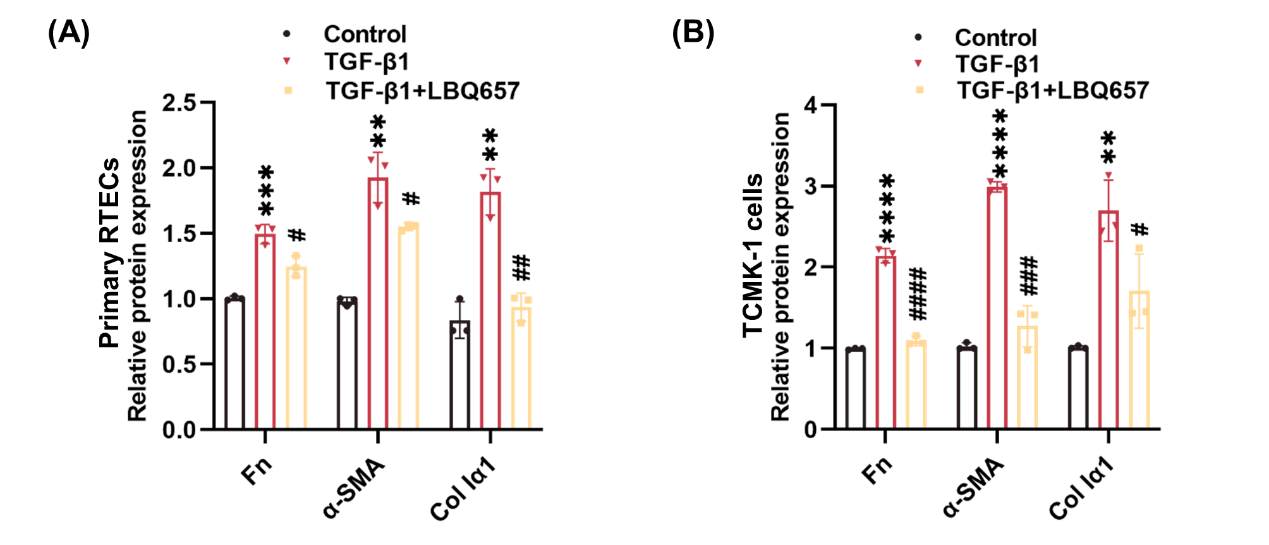
**

**FIGURE S6** **Neprilysin inhibition alleviated fibrotic phenotype in TGF-β1-stimulated renal tubular epithelial cells.** (A) Semi-quantification of Western blot was performed to detect protein expression of Fn, α-SMA, and Col Iα1 in primary renal tubular epithelial cells (RTECs). (B) Semi-quantification of Western blot was performed to detect protein expression of Fn, α-SMA, and Col Iα1 in TCMK-1 cells. *****P <* 0.0001, ****P <* 0.001, ***P <* 0.01, versus Control. ^####^*P <* 0.0001, ^###^*P <* 0.001, ^##^*P <* 0.01, ^#^*P <* 0.05, versus TGF-β1.

**
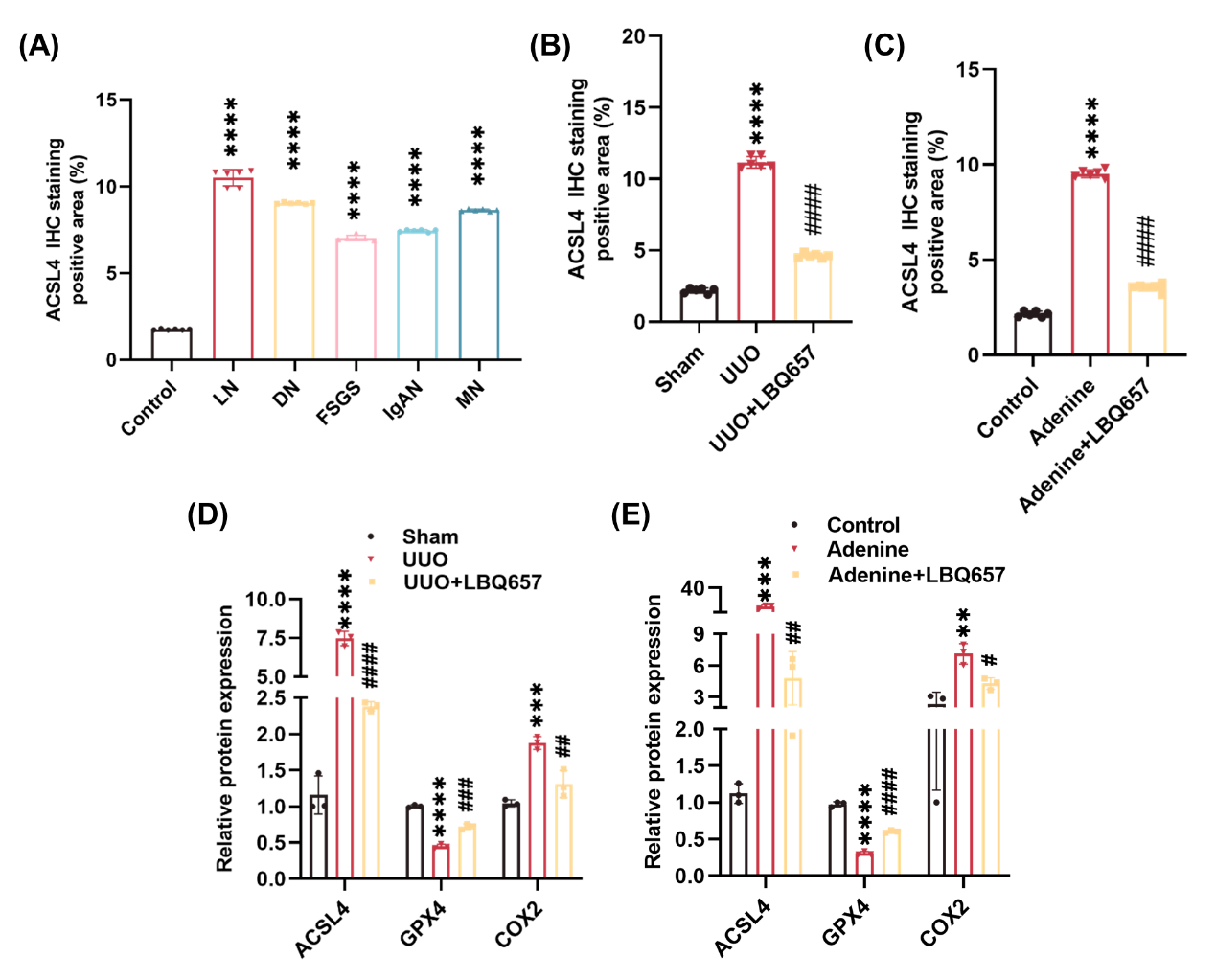
**

**FIGURE S7 Ferroptosis in fibrotic kidney was alleviated by inhibition of neprilysin.** (A) Percentage of ACSL4-positive area in human kidney sections. LN, lupus nephritis; DN, diabetic Nephropathy; FSGS, focal segmental glomerulosclerosis; IgAN, IgA nephropathy; MN, membranous nephropathy. (B,C) Percentage of ACSL4-positive area in mouse kidney sections. (D,E) Semi-quantification of Western blot was performed to detect protein expression of ACSL4, GPX4 and COX2. *****P<*0.0001, ****P<*0.001, ***P<*0.01, versus Sham or Control. ^####^*P<*0.0001, ^###^*P<*0.001, ^##^*P<*0.01, ^#^*P<*0.05, versus UUO or Adenine.


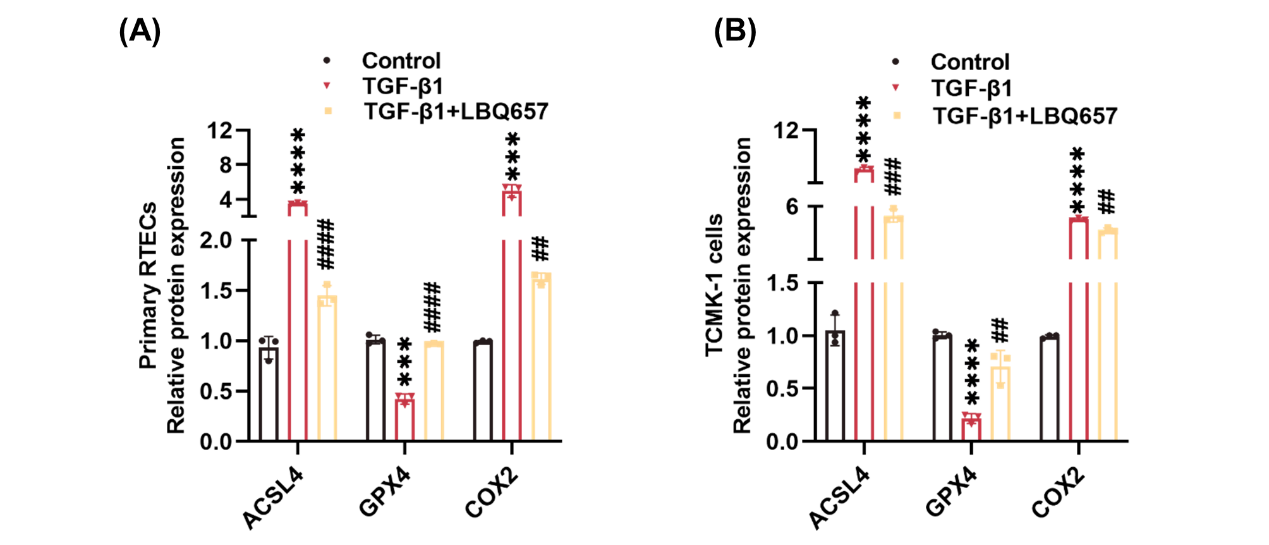


**FIGURE S8** **Pharmacological inhibition of neprilysin alleviated TGF-β1-induced ferroptosis in primary renal tubular epithelia cells (RTECs) and TCMK-1 cells.** (A) Semi-quantification of Western blot was performed to detect protein expression of ACSL4, GPX4, and COX2 in RTECs. (B) Semi-quantification of Western blot was performed to detect protein expression of ACSL4, GPX4, and COX2 in TCMK-1 cells. *****P<*0.0001, ****P<*0.001, versus Control. ^####^*P<*0.0001, ^###^*P<*0.001, ^##^*P<*0.01, versus TGF-β1.

**
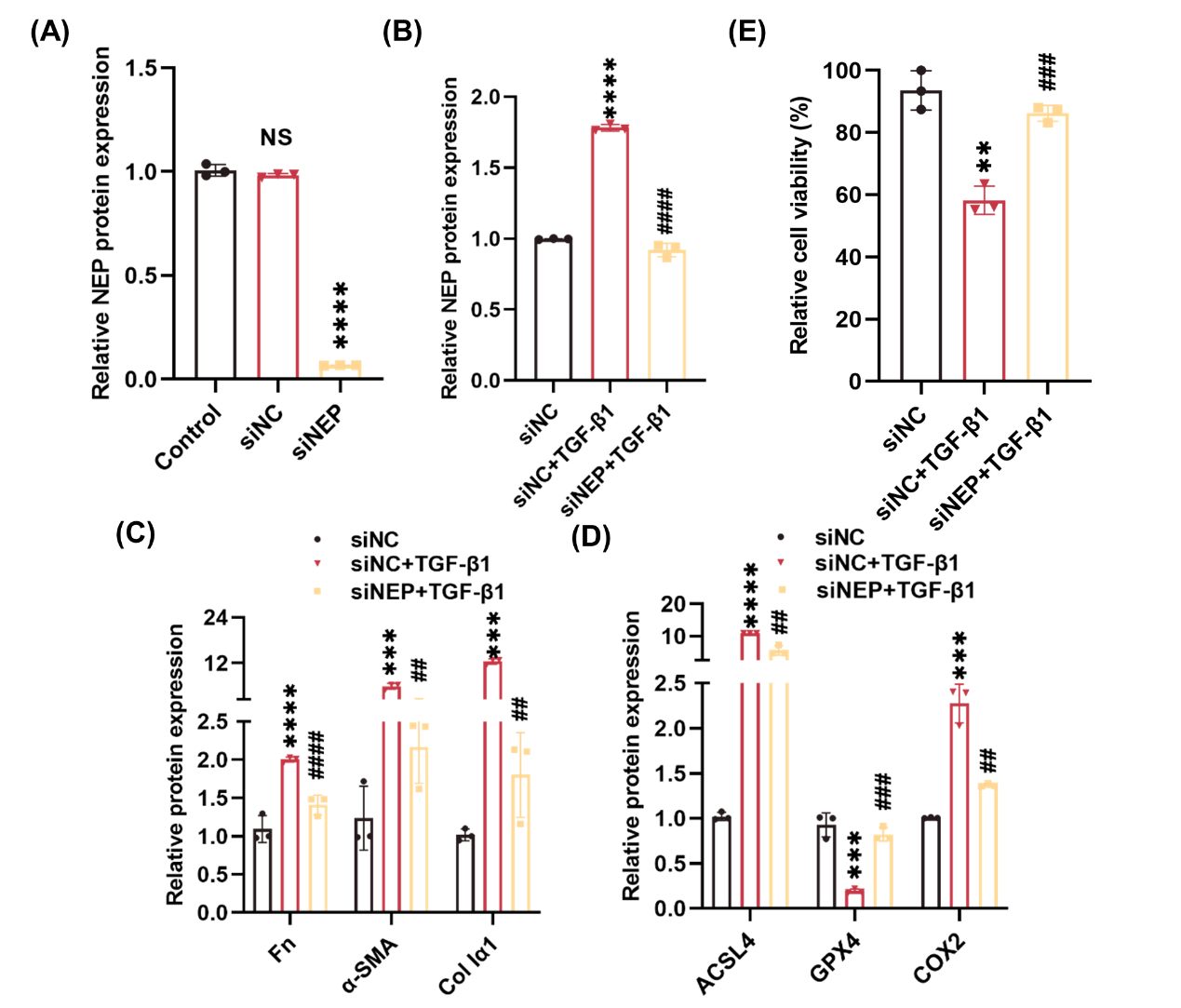
**

**FIGURE S9** **Genetic knockdown of neprilysin (NEP) improved fibrotic and ferroptotic phenotype in TGFβ1-induced TCMK-1 cells.** (A,B) Semi-quantification of Western blot was performed to detect protein expression of NEP. (C) Semi-quantification of Western blot was performed to detect protein expression of Fn, α-SMA, and Col Iα1. (D) Semi-quantification of Western blot was performed to detect protein expression of ACSL4, GPX4, and COX2. (E) Relative cell viability evaluated by the CCK-8 assay. ^NS^*P* > 0.05, versus Control. *****P <* 0.0001, ****P <* 0.001, ***P <* 0.01, versus siNC. ^####^*P <* 0.0001, ^###^*P <* 0.001, ^##^*P <* 0.01, ^#^*P <* 0.05, versus siNC+TGF-β1. siNC, negative control.

**
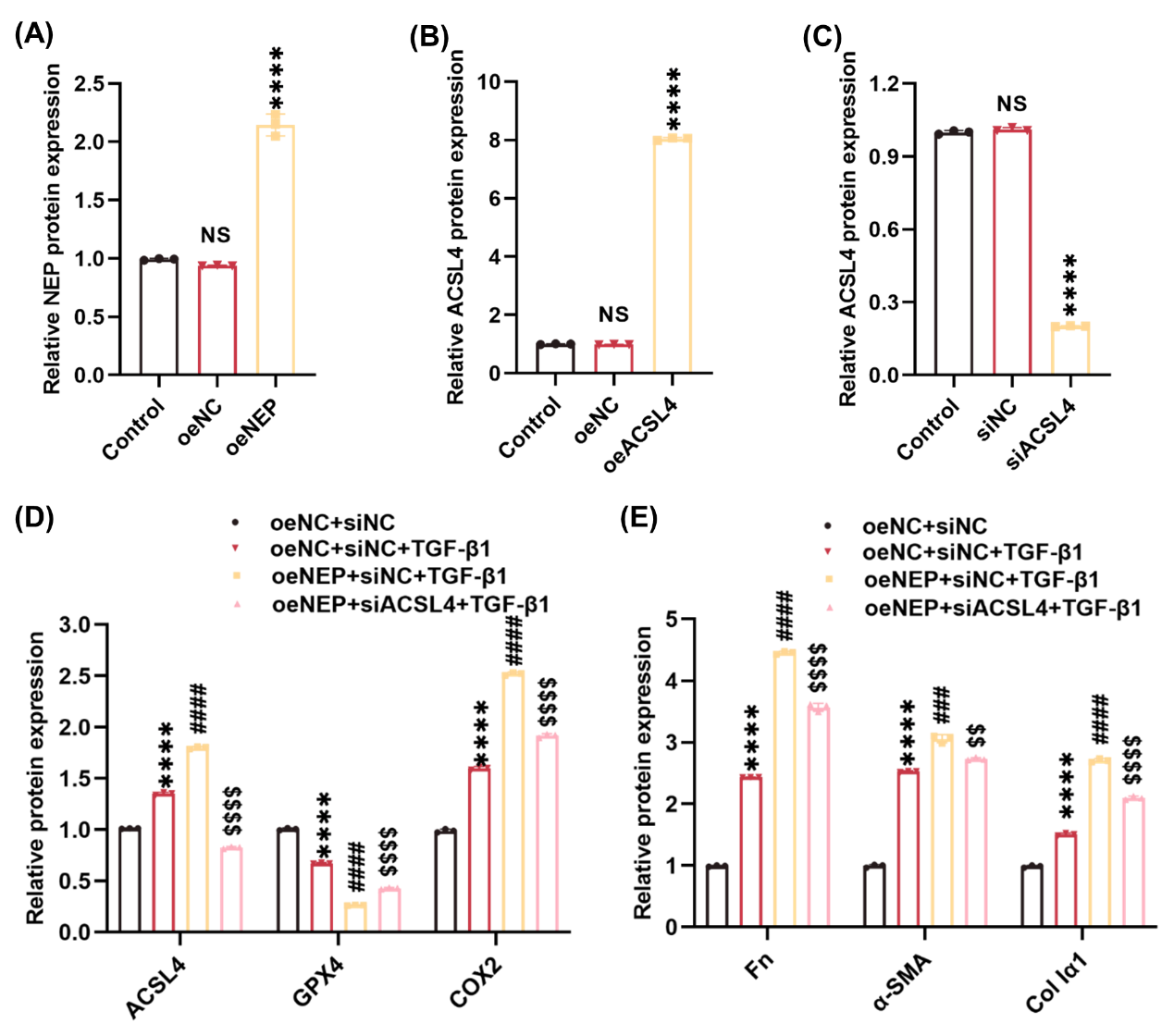
**

**FIGURE S10** **Neprilysin (NEP) aggravated fibrosis by ACSL4-mediated ferroptosis in TGF-β1-induced TCMK-1 cells.** (A-C) Semi-quantification of Western blot was performed to detect protein expression of NEP and ACSL4. (D) Semi-quantification of Western blot was performed to detect protein expression of ACSL4, GPX4, and COX2. (E) Semi-quantification of Western blot was performed to detect protein expression of Fn, α-SMA, and Col Iα1. ^NS^*P* > 0.05, versus Control. *****P <* 0.0001, versus oeNC or siNC or oeNC+siNC. ^####^*P <* 0.0001, ^###^*P <* 0.001, versus oeNC+siNC+TGF-β1. ^$$$$^*P <* 0.0001, ^$$^*P <* 0.01, versus oeNEP+siNC+TGF-β1. siNC, silencing negative control; siNEP, NEP siRNA; siACSL4, ACSL4 siRNA; oeNC, overexpression negative control; oeNEP, overexpression of NEP; oeACSL4, overexpression of ACSL4.


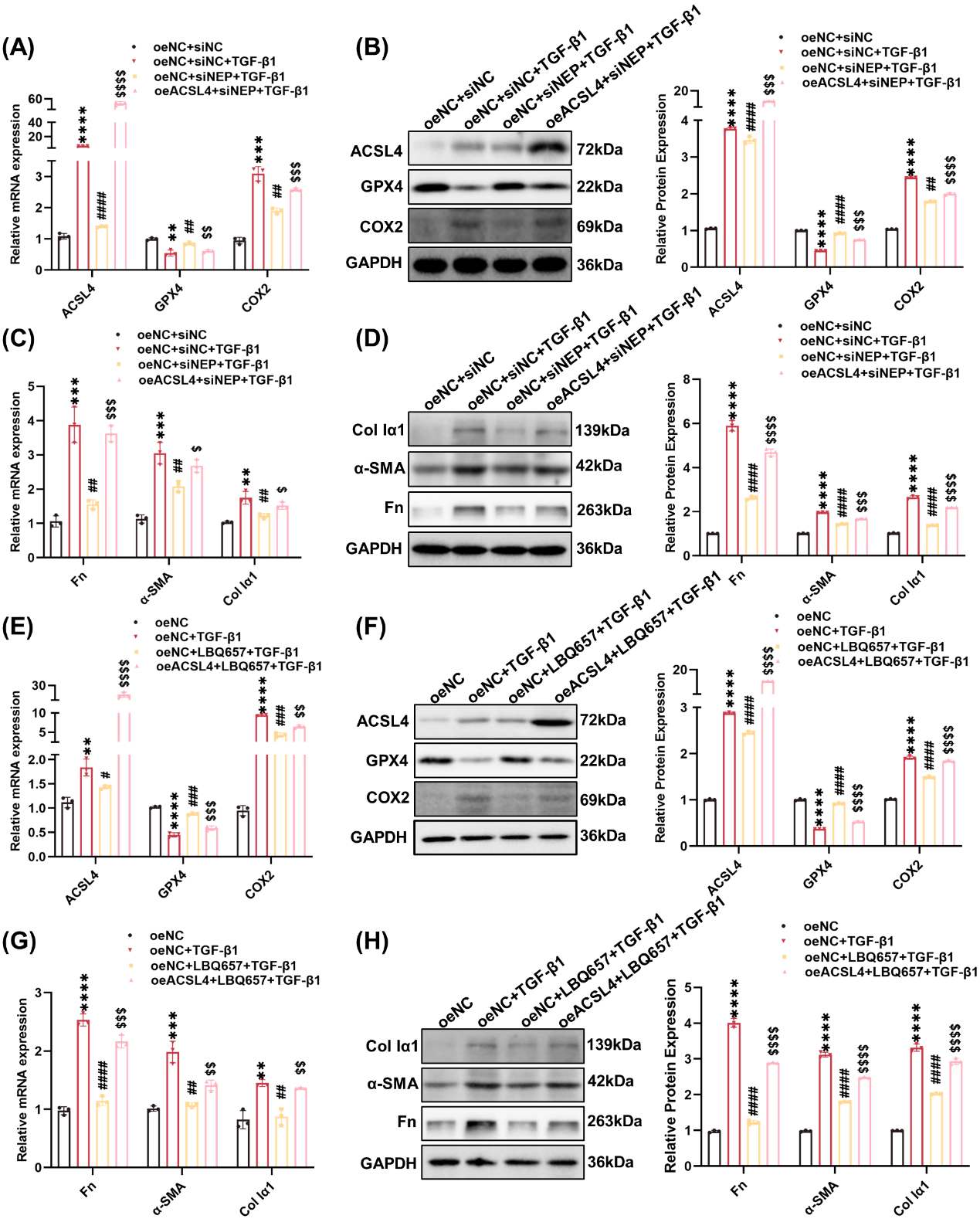


**FIGURE S11** **Neprilysin (NEP) inhibition attenuated fibrosis by downregulating ACSL4-meidated ferroptosis in TGF-β1-induced TCMK-1 cells.** (A,E) RT-PCR was performed to detect mRNA level of ACSL4, GPX4, and *Ptgs2* (COX2). (B,F) Western blot analysis was performed to detect the protein expression of ACSL4, GPX4, and COX2. (C,G) RT-PCR was performed to detect mRNA level of Fn, α-SMA, and Col Iα1. (D,H) Western blot analysis was performed to detect protein expression of Fn, α-SMA and Col Iα1. *****P <* 0.0001, ****P <* 0.001, ***P <* 0.01, versus oeNC or oeNC+siNC. ^####^*P <* 0.0001, ^###^*P <* 0.001, ^##^*P <* 0.01, ^#^*P <* 0.05, versus oeNC+TGF-β1 or oeNC+siNC+TGF-β1. ^$$$$^*P <* 0.0001, ^$$$^*P <* 0.001, ^$$^*P <* 0.01, ^$^*P <* 0.05, versus oeNC+siNEP+TGF-β1 or oeNC+LBQ657+TGF-β1. siNC, silencing negative control; siNEP, NEP siRNA; siACSL4, ACSL4 siRNA; oeNC, overexpression negative control; oeNEP, overexpression of NEP; oeACSL4, overexpression of ACSL4.


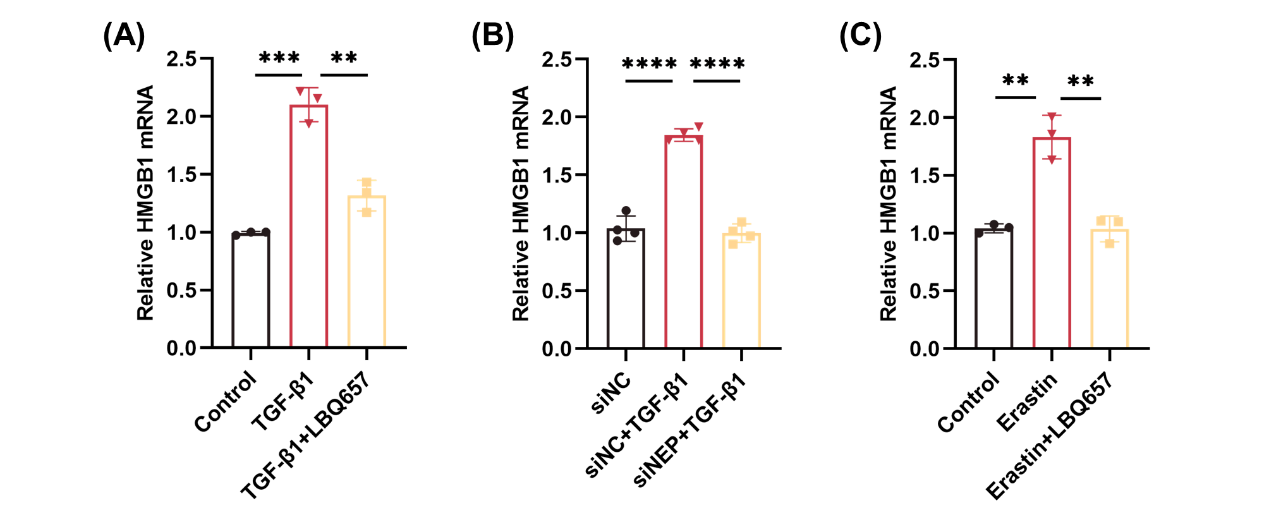


**FIGURE S12** **Neprilysin (NEP) inhibition reduced the secretion of high mobility group box 1 (HMGB1) in ferroptotic TCMK-1 cells.** (A,B) RT-PCR was performed to detect the mRNA level of HMGB1 in TGF-β1-induced TCMK-1cells. (C) RT-PCR was performed to detect the mRNA level of HMGB1 in erastin (4 μM)-induced TCMK-1 cells. *****P <* 0.0001, ****P <* 0.001, ***P <* 0.01.


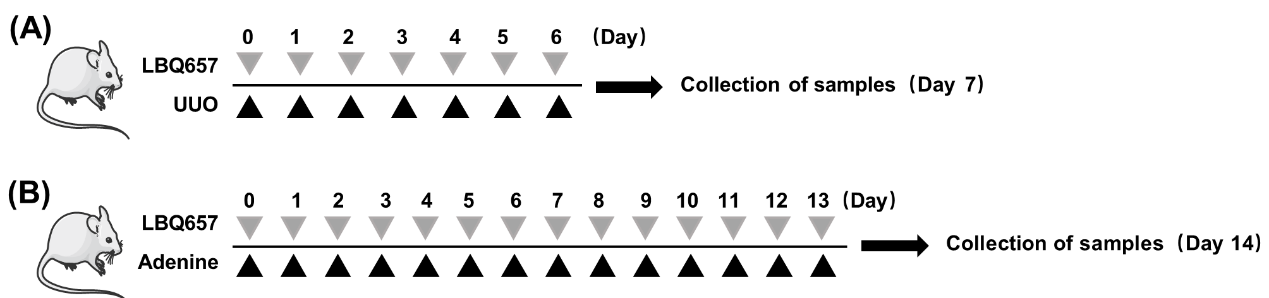


**FIGURE S13** **Schematic representation of the dosing regimen.** (A) Dosage of *Sacubitrilat* (LBQ657) administration in UUO mouse model. (B) Dosage of LBQ657 administration in adenine diet-induced mouse model.

**TABLE S1** **Primary antibodies**

| **Name** | **Company** | **Cat No.** |
| --- | --- | --- |
| Rabbit Anti-NEP | Proteintech, USA | 18008-1-AP |
| Rabbit Anti-Fibronectin | Boster, China | BA1772 |
| Rabbit Anti-Collagen Iα1 | Boster, China | BM4017 |
| Rabbit Anti-Collagen VI | Huabio, China | ET1612-91 |
| Rabbit Anti-α-SMA | Huabio, China | ET1607-43 |
| Mouse Anti- GAPDH | Abcam, USA | EM1101 |
| Mouse Anti-IL-6 | Huabio, China | EM170414 |
| Rabbit Anti-IL-1β | Affinity Biosciences, USA | AF4006 |
| Rabbit Anti-TNF-α | Affinity Biosciences, USA | AF7014 |
| Rabbit Anti-MCP-1 | Affinity Biosciences, USA | AF7577 |
| Goat anti-KIM1 | R&D Systems, USA | AF1817 |
| Rabbit anti-NGAL | Affinity Biosciences, USA | DF6816 |
| Rabbit anti-ACSL4 | Proteintech, USA | 81196-1-RR |
| Rabbit anti-GPX4 | Huabio, China | ET1706-45 |
| Rabbit anti-COX2 | Proteintech, USA | 27308-1-AP |
| Horseradish labeled goat anti-mouse IgG | Huabio, China | HA1006 |
| Horseradish labeled alpaca anti-rabbit IgG | Huabio, China | HA1031 |

NEP, neprilysin; α-SMA, α-smooth muscle actin; MCP-1, monocyte chemotactic protein 1; TNF-α, tumor necrosis factor-α; IL, Interleukin; KIM-1, kidney injury molecule 1; NGAL, neutrophil gelatinase-associated lipocalin; ACSL4, acyl-CoA synthetase long-chain family member 4; GPX4, glutathione peroxidase 4; COX2, cyclooxygenase 2.

**TABLE S2** **Primers sequences for RT-PCR**

| **Gene** | **Source** | **Sequences（5’ -3’）** |
| --- | --- | --- |
| MME (NEP) | Mouse | CTCTCTGTGCTTGTCTTGCTC  GACGTTGCGTTTCAACCAGC |
| Fibronectin | Mouse | GCAAACCTATAGCTGAGAAGTG  CAAGTACAGTCCACCATCATC |
| α-SMA | Mouse | TCTCAAACATAATCTGGGTCA  CAGGGAGTAATGGTTGGAAT |
| Collagen Iα1 | Mouse | TGCCGCGACCTCAAGATGTG  CACAAGGGTGCTGTAGGTGA |
| Collagen IVα1 | Mouse | CTGGCACAAAAGGGACGAG  ACGTGGCCGAGAATTTCACC |
| Collagen VI α1 | Mouse | CTGCTGCTACAAGCCTGCT  GCACGAAGAATAGATCCACAGGG |
| GAPDH | Mouse | GTATGACTCCACTCACGGCAAA  GGTCTCGCTCCTGGAAGATG |
| MCP-1 | Mouse | CATCCACGTGTTGGCTCA  GATCATCTTGCTGGTGAATGAGT |
| TNF-α | Mouse | ACCCTCACACTCAGATCATCTTC  TGGTGGTTTGCTACGACGT |
| IL-1β | Mouse | CCTCGTGCTGTCGGACCCATA  CAGGCTTGTGCTCTGCTTGTGA |
| IL-6 | Mouse | ACAACCACGGCCTTCCCTACTT  CACGATTTCCCAGAGAACATGTG |
| Havcr1(KIM-1) | Mouse | ACATATCGTGGAATCACAACGAC  ACTGCTCTTCTGATAGGTGACA |
| Lcn2 (NGAL) | Mouse | GCAGGTGGTACGTTGTGGG  CTCTTGTAGCTCATAGATGGTGC |
| ACSL4 | Mouse | CTCACCATTATATTGCTGCCTGT  TCTCTTTGCCATAGCGTTTTTCT |
| GPX4 | Mouse | GCCTGGATAAGTACAGGGGTT  CATGCAGATCGACTAGCTGAG |
| Ptgs2 (COX2) | Mouse | TGAGCAACTATTCCAAACCAGC  GCACGTAGTCTTCGATCACTATC |

NEP, neprilysin; α-SMA, α-smooth muscle actin; MCP-1, monocyte chemotactic protein 1; TNF-α, tumor necrosis factor-α; IL, Interleukin; KIM-1, kidney injury molecule 1; NGAL, neutrophil gelatinase-associated lipocalin; ACSL4, acyl-CoA synthetase long-chain family member 4; GPX4, glutathione peroxidase 4; COX2, cyclooxygenase 2.

**TABLE S3 The siRNA sequences for transfection**

| **Gene** | **Source** | **siRNA Sequence （5’ -3’）** |
| --- | --- | --- |
| NEP | Mouse | GCCUAUUCUUACCAAAUAUTT  AUAUUUGGUAAGAAUAGGCTT |
| ACSL4 | Mouse | CCUGUCCACUUGUUAAUAATT  UUAUUAACAAGUGGACAGGTT |

NEP, neprilysin；ACSL4, acyl-CoA synthetase long-chain family member 4.
